# Advancements in Photovoltaic Cell Materials: Silicon, Organic, and Perovskite Solar Cells

**DOI:** 10.3390/ma17051165

**Published:** 2024-03-01

**Authors:** Abniel Machín, Francisco Márquez

**Affiliations:** 1 Environmental Catalysis Research Laboratory, Division of Natural Sciences and Technology, Universidad Ana G. Méndez-Cupey Campus, San Juan, PR 00926, USA; 2 Nanomaterials Research Group, Department of Natural Sciences and Technology, Universidad Ana G. Méndez-Gurabo Campus, Gurabo, PR 00778, USA

**Keywords:** photovoltaic cells, silicon-based solar cells, organic-based cells, perovskite solar cells

## Abstract

The evolution of photovoltaic cells is intrinsically linked to advancements in the materials from which they are fabricated. This review paper provides an in-depth analysis of the latest developments in silicon-based, organic, and perovskite solar cells, which are at the forefront of photovoltaic research. We scrutinize the unique characteristics, advantages, and limitations of each material class, emphasizing their contributions to efficiency, stability, and commercial viability. Silicon-based cells are explored for their enduring relevance and recent innovations in crystalline structures. Organic photovoltaic cells are examined for their flexibility and potential for low-cost production, while perovskites are highlighted for their remarkable efficiency gains and ease of fabrication. The paper also addresses the challenges of material stability, scalability, and environmental impact, offering a balanced perspective on the current state and future potential of these material technologies.

## 1. Introduction

The journey of photovoltaic (PV) cell technology is a testament to human ingenuity and the relentless pursuit of sustainable energy solutions. From the early days of solar energy exploration to the sophisticated systems of today, the evolution of PV cells has been marked by groundbreaking advancements in materials and manufacturing processes. The initial phase of solar cell development was characterized by the use of crystalline silicon, a material that has maintained its prominence due to its proven efficiency and durability [1]. The progression from the initial 15% efficiency in the 1950s to the current levels nearing 28% epitomizes the significant strides that have been made in enhancing solar cell performance [2]. This evolution is a clear indicator of how material advancements have been instrumental in propelling the solar industry forward.

The significance of material advancements in solar cell technology extends beyond mere efficiency improvements. In the context of escalating environmental concerns and the global imperative for renewable energy sources, solar energy emerges as a beacon of hope. The notable reduction in solar energy generation costs over the past decade is a direct consequence of advancements in materials, alongside innovations in technology and enhanced panel efficiencies [1]. The pursuit of new materials, novel concepts, and innovative approaches in solar cell development is central to achieving high efficiencies at reduced costs. This endeavor is not just about enhancing technology; it is about democratizing access to solar energy, making it a feasible option for a broader segment of the global population [3].

With that in mind, this review aims to provide an analysis of the advancements in photovoltaic cell materials, with a particular focus on silicon-based, organic, and perovskite solar cells. Each of these materials bring unique attributes and challenges to the table, collectively shaping the current and future landscape of solar energy technology. The review will delve into the historical context of these materials, explore recent innovations, and project future prospects. It will offer insights into their efficiency, commercial viability, and environmental implications. A comparative analysis across these material classes will shed light on their efficiency, stability, and scalability, underscoring the specific challenges and potential solutions that are inherent to each type. In addition to a technical analysis, the review will address the broader implications of these material advancements. Topics such as material stability, scalability, manufacturing techniques, and the environmental impact of solar cell production will be thoroughly examined. The objective is to present a holistic view of the current state of PV technology, while also identifying emerging trends and potential breakthroughs that could significantly influence the future of solar energy. Furthermore, policy and market dynamics will be discussed, exploring the potential of integrating solar cells into the global energy mix and the factors that will drive their widespread adoption.

## 2. Silicon-Based Solar Cells

### 2.1. Historical Context and Enduring Relevance

Silicon-based solar cells have not only been the cornerstone of the photovoltaic industry for decades but also a symbol of the relentless pursuit of renewable energy sources. The journey began in 1954 with the development of the first practical silicon solar cell at Bell Labs, marking a pivotal moment in the history of solar energy [4]. This invention, achieving an efficiency of about 6%, was a significant leap from earlier solar energy attempts, which were largely inefficient and impractical for widespread use [4].

The dominance of silicon in the photovoltaic market can be attributed to several key factors. Firstly, silicon is the second most abundant element in the Earth’s crust, making it readily available for solar cell production [5]. This abundance has been a critical factor in the widespread adoption and scalability of silicon-based solar cells. Secondly, the semiconductor properties of silicon make it an ideal material for converting sunlight into electricity. Its bandgap is well suited for absorbing a broad range of the solar spectrum, thereby maximizing the energy conversion efficiency [5].

Over the years, the manufacturing processes for silicon solar cells have undergone significant evolution, transitioning from simple p-n junctions to more complex designs that enhance light absorption and minimize energy losses [6]. The development of crystalline silicon technology, both in monocrystalline and polycrystalline forms, has been central to this evolution. Monocrystalline silicon cells, known for their higher efficiency due to their uniform crystalline structure, have become increasingly popular in high-performance applications [6]. On the other hand, polycrystalline silicon cells, made from multiple silicon crystals, offer a more cost-effective solution, albeit with slightly lower efficiency [6].

The 1970s and 1980s were marked by significant milestones in the development of silicon-based solar cells, with the introduction of new technologies such as surface passivation and antireflective coatings [7]. These innovations were crucial in enhancing the efficiency and durability of silicon solar cells, propelling them to the forefront of solar energy solutions. By the late 20th century, silicon solar cells had firmly established themselves as the standard in the photovoltaic industry, with efficiencies surpassing 15% [7].

In the 21st century, the focus shifted towards further improving the efficiency and reducing the cost of silicon solar cells. The introduction of PERC (passivated emitter and rear cell) technology and the development of bifacial solar cells are examples of innovations that have significantly boosted the performance of silicon-based solar cells [8]. These advancements have not only improved efficiency but also extended the lifespan of solar panels, making them more appealing for both residential and commercial applications [8].

### 2.2. Recent Innovations in Crystalline Silicon Structures

Regarding crystalline silicon (c-Si) solar cells, recent years have been marked by groundbreaking innovations aimed at transcending the traditional efficiency limits. These advancements are pivotal in sustaining silicon’s competitiveness in the rapidly evolving photovoltaic market. A notable example is the work by Zhang et al. [9], which delves into the realm of perovskite/crystalline silicon tandem solar cells. Their research systematically reviews the latest progress in this area, focusing on the structure of perovskite top cells, intermediate interconnection layers, and crystalline silicon bottom cells. They emphasize the importance of optical and electrical engineering in each layer, highlighting how these aspects are integral throughout the device preparation process. This study is significant, as it demonstrates the potential of tandem cells to achieve efficiencies above 30%, a remarkable feat in solar cell technology [9].

**Figure 1:**
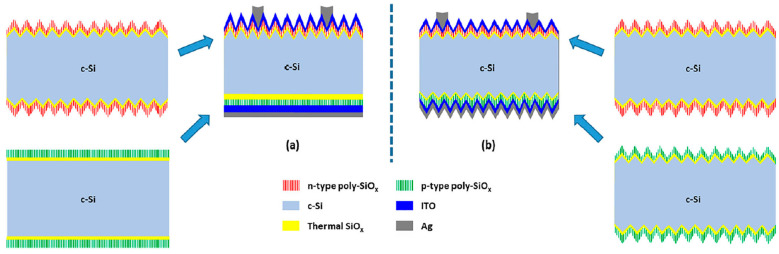
(**a**) A single-sided textured (SST) poly-SiOx passivated crystalline silicon (c-Si) solar cell, featuring symmetric n-type doped poly-SiOx on a double-sided textured (DST) substrate at the top and symmetric p-type doped poly-SiOx on a double-sided polished (DSP) substrate at the bottom; (**b**) a DST poly-SiOx passivated c-Si solar cell, equipped with symmetric n-type doped poly-SiOx on the top and symmetric p-type doped poly-SiOx, both situated on DST substrates. “ITO” refers to indium tin oxide. Reprinted with permission from ref. [10], copyright 2023, Progress in Photovoltaics: Research and Applications.

Another significant contribution comes from Singh et al. [10] and presents the creation of c-Si bottom cells using high-temperature polycrystalline-SiOx (poly-SiOx) carrier-selective passivating contacts (CSPCs), a promising approach for high-efficiency tandem cells (see Figure 1). The research involved tuning ultra-thin SiOx layers and optimizing the passivation of both p-type and n-type doped poly-SiOx CSPCs, with a focus on p-type doped poly-SiOx CSPCs on textured interfaces through a two-step annealing process. The integration of these optimized bottom cells into four-terminal (4T) and two-terminal (2T) tandem structures led to a conversion efficiency of 28.1% and 23.2%, respectively.

Furthering the innovation in thin crystalline silicon solar cells, the study by Xie et al. [11] reported significant advancements in the efficiency of thin crystalline silicon (c-Si) solar cells, a promising alternative to the traditional, thicker c-Si solar cells, due to their cost-effectiveness and enhanced flexibility. Their approach involved the implementation of advanced cell design optimizations, focusing on a prototype with a thickness of 20 μm. The results of their optimizations are notable: the short-circuit current density increased from 34.3 mA/cm^2^ to 38.2 mA/cm^2^, the open-circuit voltage improved from 632 mV to 684 mV, and the fill factor exhibited an enhancement from 76.2% to 80.8%. These improvements collectively resulted in a significant absolute efficiency increase of 4.6%, elevating the overall efficiency from 16.5% to 21.1%. The experimental outcomes were corroborated by device simulations, providing a comprehensive understanding of the efficiency enhancements that can be achieved through optimized design strategies.

Additionally, the work by Yamamoto et al. [12] presents a 29.2% power conversion efficiency in a two-terminal (2T) perovskite/crystalline Si hetero-junction tandem solar cell, using a 145 μm thick industrial Czochralski (CZ) Si wafer. This achievement, a notable advancement in 2T tandem solar cell technology, is primarily due to structural optimizations like improved surface passivation of the perovskite layer and advanced light management techniques. Addressing the industrial application challenges, the authors also explored the potential of four-terminal (4T) tandem solar cells as a viable alternative. Drawing on their foundational technologies, which have already achieved a 22.2% efficient perovskite single-junction solar cell module and a 26% efficient hetero-junction back contact solar cell, they demonstrated the feasibility of achieving an around 30% conversion efficiency in 4T perovskite/hetero-junction crystalline Si tandem solar cells, with a significantly reduced cell size of approximately 64 cm^2^.

### 2.3. Efficiency and Commercial Viability Analysis

The efficiency of silicon-based solar cells has seen a remarkable increase over the years, with commercial monocrystalline silicon solar cells now achieving efficiencies of over 20% [13]. This improvement is largely attributed to the incorporation of advanced materials and innovative cell designs. A significant contribution to this advancement is the widespread adoption of passivated emitter and rear cell (PERC) technology, which offers higher efficiency and lower production costs compared to traditional c-Si cells [13].

J. Müller’s work [13] highlights the significant improvements in cost reduction and conversion efficiency increase that have been achieved in large-scale industrial production over the last decade. This progress has made photovoltaics (PVs) cost-competitive with other electricity generation methods. Müller discusses the key concepts and methods based on Hanwha Q CELLS’ experience, including the fast transfer of cell technologies from laboratory to production and accelerated progress in cell efficiency, quality, and reliability. The study notes that the cell conversion efficiency has increased by 0.5% abs per year, with average cell conversion efficiencies exceeding 20% using boron-doped p-type multicrystalline (mc-Si) substrates and 22% using Czochralski-grown silicon (Cz-Si) substrates [13].

In an effort to reduce the cost of photovoltaic (PV) power generation, Irie and group [14] focused on three primary objectives: lowering the manufacturing costs of PV modules, improving the efficiencies of cells and modules, and extending the long-term output power warranty of PV modules. They developed a high-quality and cost-effective seed-cast wafer, which achieved an efficiency of 20.54% with passivated emitter and rear cells (PERCs). Additionally, the authors addressed module longevity concerns by identifying and mitigating key degradation modes, including ohmic contact degradation and potential induced degradation (PID). To assess the durability of their modules under real-world conditions, they conducted extensive stress tests, simulating environments with ultraviolet light, heat, humidity, and electrical potential differences. These tests, including those on field-aged modules, demonstrated that their technology can ensure a module lifetime exceeding 30 years, with resistance to PID, particularly in the context of Japanese domestic environments, marking a significant advancement in PV module technology.

**Figure 2:**
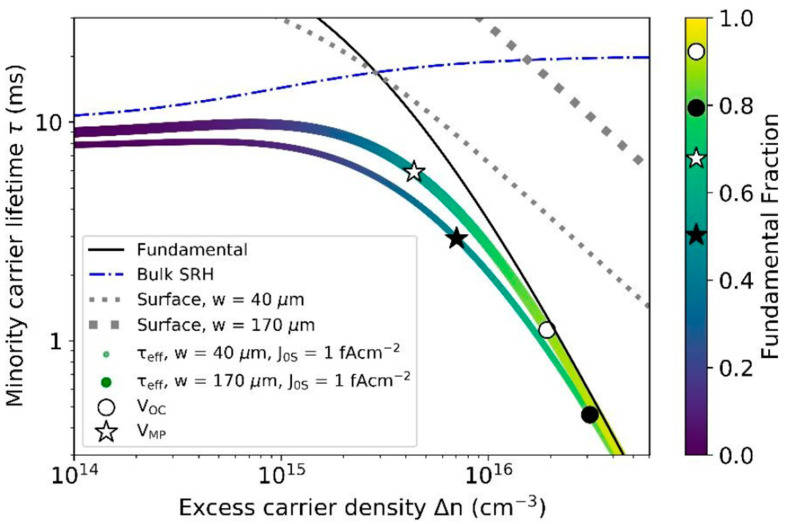
Model for the effective minority carrier lifetime in structures using n-type wafers of thicknesses of 170 μm and 40 μm. These wafers are characterized by a bulk Shockley–Read–Hall (SRH) lifetime of 10 ms, a bulk resistivity of 3.55 Ω cm (equivalent to a dopant concentration of 1.3 × 10^15^ cm⁻^3^), and a combined surface recombination velocity (J0S) of 1 fAcm⁻^2^ from both surfaces. In the accompanying plots, various curves are used to distinguish between different recombination mechanisms. A color bar is included to denote the proportion of fundamental recombination, which encompasses both Auger and radiative processes. The black star represents the maximum voltage at a close circuit, whereas the black circle represents the power voltage at a close circuit. The generation current for these wafers is determined based on the appropriate Lambertian light trapping limit for each specific thickness. Additionally, markers within the color bar highlight the proportions of fundamental recombination at the points of maximum power and during open-circuit injections. Reprinted with permission from ref. [15], copyright 2020, Journal of Materials Chemistry A.

Augusto and colleagues [15] reported significant advancements in silicon solar cell technologies, with several technologies now surpassing or nearing 26% efficiency. This progress is largely due to the integration of dielectric and amorphous silicon-based passivation layers and the reduction in metal/silicon contact areas, leading to surface saturation current densities below 3 fA cm⁻^2^ (see Figure 2). They found that in passivated contact solar cells, the majority of the recombination at open circuit is due to fundamental processes like Auger and radiative recombination, accounting for over three-quarters of the total recombination. However, this fraction decreases significantly at the maximum power point, where surface and bulk Shockley–Read–Hall recombination mechanisms become prevalent. Their study emphasizes the importance of reducing bulk recombination and enhancing surface passivation to improve solar cell performance under operational conditions. The authors demonstrated that thinner wafers and lower surface saturation current densities below 1 fA cm⁻^2^ are crucial for increasing the practical efficiency limit by up to 0.6% absolute. For high-quality n-type bulk silicon with a minority carrier lifetime of 10 ms, they identified an optimal wafer thickness range of 40–60 μm, which was significantly different to the previously assumed 110 μm. Within this thickness range, achieving surface saturation current densities near 0.1 fA cm⁻^2^ is essential to approach the fundamental efficiency limit. Experimentally, they have achieved surface saturation currents below 0.5 fA cm⁻^2^ on pi/CZ/in structures across a range of wafer thicknesses (35–170 μm), indicating the potential to attain open-circuit voltages close to 770 mV and bandgap–voltage offsets near 350 mV. Finally, the authors suggest using the bandgap–voltage offset as a comparative metric for evaluating the quality of high-performing experimental solar cells across various commercially relevant photovoltaic cell absorbers and architectures.

Mao’s research [16] explores the dominance and evolution of crystalline silicon solar cells in the photovoltaic market, focusing on the transition from polycrystalline to more cost-effective monocrystalline silicon cells, which is driven by advancements in silicon materials and wafer technologies. The study highlights the increasing conversion efficiency of monocrystalline cells, particularly through high-efficiency technologies like passivated emitter and rear cells (PERCs). They analyzed and forecast the future of solar cell industrialization, concluding that N-type tunnel oxide passivated contact (TOPCon) solar cell technology is poised to become the next mainstream technology after PERC. Additionally, the authors identified interdigitated back contact (IBC) structures and selective all-passive contact technologies as viable paths to achieving high-efficiency solar cells. This synthesis of efficiency, cost, and technological compatibility underlines the potential for the industrialization of cost-effective, high-efficiency monocrystalline silicon solar cells.

In terms of commercial viability, silicon solar cells continue to benefit from economies of scale and well-established supply chains. The cost of silicon PV cells has decreased significantly, making solar energy more competitive with traditional energy sources. However, the market also faces challenges such as the need for more sustainable manufacturing processes and the management of end-of-life solar panels.

### 2.4. Challenges and Future Outlook

Despite their success, silicon-based solar cells face several challenges. One of the primary challenges is the nearing of the theoretical efficiency limit for single-junction silicon cells [17]. This limitation has necessitated the exploration of new designs, such as tandem cells. Rong et al. [17] review the progress in perovskite solar cells (PSCs), which are increasingly being considered for tandem applications with silicon cells. They note that PSCs have achieved lab-scale power conversion efficiencies of 23.3%, rivaling commercial multicrystalline silicon solar cells. However, stability and upscaling for mass production remain critical concerns for the commercialization of PSCs [17].

Environmental concerns associated with the production and disposal of silicon PV cells are also significant challenges. Lunardi et al. [18] examined the expanding role of solar photovoltaics (PVs) as a sustainable and low-carbon electricity source, focusing on a life cycle assessment (LCA) of current and emerging solar cell technologies, predominantly silicon wafer cells and prospective silicon/thin-film tandem devices. They demonstrated that efficiency enhancements, especially through the integration of atomic hydrogen in silicon wafers, offer significant environmental benefits, justifying the additional required inputs. The study also underscores the importance of top-cell material stability in tandem solar cells to prolong the lifespan of the underlying silicon bottom cell. Addressing the end-of-life scenario for PV modules, traditionally destined for landfills, the authors highlight the urgent need for sustainable recycling practices in light of the rapid global adoption of photovoltaics. Despite challenges in the environmentally and financially viable dismantling of PV modules, their research is directed towards developing effective recycling methods, including chemical, thermal, and mechanical techniques, to optimize material recovery and foster sustainable industry practices.

**Figure 3:**
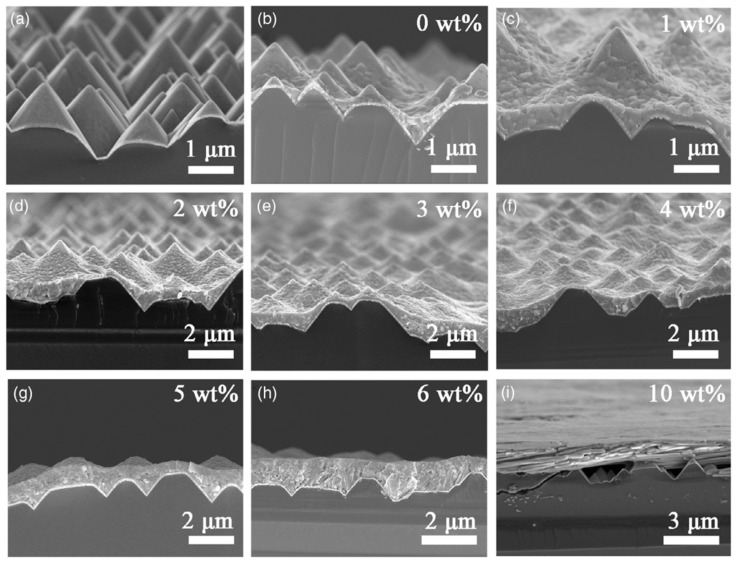
Cross-sectional scanning electron microscopy (SEM) images illustrating (**a**) a silicon surface textured with pure pyramids and (**b**–**i**) the same pyramid-textured silicon surface overlaid with perovskite films, each with varying starch ratios. These ratios include (**b**) 0 wt%, (**c**) 1 wt%, (**d**) 2 wt%, (**e**) 3 wt%, (**f**) 4 wt%, (**g**) 5 wt%, (**h**) 6 wt%, and (**i**) 10 wt%. Reprinted with permission from ref. [19], copyright 2020, Energy Technology.

Wang et al. [19] introduce a simple solvent engineering technique involving the use of starch additive in a MAPbI3-based one-step spin-coating process at room temperature, aimed at efficiently depositing perovskite on textured silicon surfaces for perovskite/Si monolithic tandem solar cells (TSCs) (see Figure 3). The authors investigate the influence of different starch concentrations on the morphological, structural, optical, and photovoltaic properties of the perovskite films. The results show that starch improved the solution viscosity and formed hydrogen bonds with CH_3_NH_3_^⁺^, facilitating the formation of perovskite films with a crystal structure that is compatible with textured silicon surfaces. A concentration of 5 wt% starch enables complete coverage of textured silicon surfaces with an average film thickness of around 600 nm. This approach not only stabilizes the crystal structure and device performance of the perovskite film and the planar solar cell but also locks water molecules at the perovskite grain boundaries due to the presence of starch. Their findings demonstrated the potential of this method in achieving uniform light absorption in perovskite layers and a well-matched current density in perovskite/Si monolithic TSCs, with a best calculated cell efficiency exceeding 29%.

Zhao et al. [20] provide a detailed exploration of recent advances in photovoltaic technologies, specifically focusing on organic and perovskite-based solar cells. The authors explore the intricacies of crystallization mechanisms in these cells, underlining their crucial role in influencing cells’ efficiency and performance. Their work also addresses the pivotal challenge of material stability, highlighting innovative approaches in charging materials to boost solar cell efficiency and durability. Additionally, it explores the commercial potential of these technologies, emphasizing scalable fabrication techniques and the promising capabilities of tandem solar cells, which are capable of exceeding the traditional Shockley–Queisser limit, thus heralding a new era in solar energy technology.

The future outlook for silicon-based solar cells is promising, with ongoing research focused on enhancing their efficiency and reducing costs. Innovations such as the integration of perovskite layers with silicon to create tandem cells, and the use of nanotechnology for light management, are expected to play a significant role in the next generation of silicon PV cells. Moreover, the industry is moving towards more sustainable practices, including the use of greener materials and the development of efficient recycling methods for solar panel components.

## 3. Gallium Arsenide (GaAs)

Gallium arsenide (GaAs) solar cells are among the highest efficiency solar cells available today. Unlike silicon-based solar cells, GaAs cells can convert more of the solar spectrum into electricity [21]. This is primarily due to the direct bandgap of GaAs, which allows for efficient absorption of sunlight and its conversion into electrical energy [21]. The highest efficiency of GaAs solar cells has surpassed 29%, a benchmark set in controlled laboratory conditions [21]. This high efficiency is attributed to the material’s ability to operate effectively at higher temperatures and its superior response to low-light conditions compared to silicon cells.

The unique properties of GaAs solar cells make them particularly suitable for space applications. Their high efficiency, coupled with a resistance to radiation, ensures long-term operation in the harsh environment of space [22]. NASA has utilized GaAs solar cells in various missions, appreciating their reliability and superior performance [22]. Furthermore, in concentrated photovoltaic (CPV) systems, GaAs solar cells are used to harness sunlight that is concentrated onto the cells by lenses or mirrors. This application benefits from the high efficiency of GaAs cells under intense light conditions, making them ideal for CPV installations where space is at a premium and maximum power output is desired [23]. Schön and group [24] present the development of thin, lightweight, flexible solar cells with an epitaxy thickness of about 10 μm, utilizing an inverted metamorphic triple-junction structure with Ga_0.51_In_0.49_P/GaAs/Ga_0.73_In_0.27_As subcells. Optimized for end-of-life (EOL) efficiency post 1 MeV electron irradiation, the cells demonstrated a predicted efficiency potential of 30.9% under AM0 illumination before irradiation and 26.7% after. The tested cells achieved an up to 30.2% conversion efficiency pre-irradiation and 25.4% post-irradiation, with a remarkable power-to-mass ratio reaching 3.0 W/g, marking significant advancements in solar cell technology. Additionally, the solar cells showed exceptional mechanical stability, with no performance degradation after rigorous temperature cycling, highlighting their potential for space applications.

Despite their advantages, GaAs solar cells face significant challenges that limit their widespread adoption [25]. The primary issue is their cost of production. Gallium and arsenic, the core materials in GaAs cells, are expensive and less abundant than silicon, leading to higher manufacturing costs. The complex manufacturing process of GaAs cells, which requires high-purity materials and sophisticated fabrication techniques, further escalates the cost [25]. Material scarcity is another concern [26]. Gallium is a byproduct of the smelting of other metals, notably aluminum and zinc, and its availability is dependent on the production levels of these metals. Arsenic, while more abundant, poses environmental and health risks during extraction and processing, necessitating stringent handling and disposal measures [26].

Efforts are underway to address the cost and material scarcity challenges faced by GaAs solar cells. Innovations in manufacturing techniques, such as the development of thin-film GaAs solar cells, aim to reduce the material usage and production costs [27]. Thin-film technologies allow for the deposition of GaAs layers on inexpensive substrates, significantly lowering the amount of gallium and arsenic that is required [27].

Recycling of gallium from electronic waste is another avenue being explored to mitigate material scarcity. As the demand for electronics continues to grow, so does the potential for recycling gallium, providing a more sustainable source for the production of GaAs solar cells [28]. Ndalloka et al. [29] explain that GaAs solar cells, known for their direct bandgap and high efficiencies of up to 28.8% for single-junction and 42.3% for triple-junction cells, are crucial in space and to achieve concentrated PV power generation due to their durability and thermal stability. Despite their efficiency, production sees nearly 85% of GaAs wasted, highlighting the need for advanced recycling methods. Techniques like nitrogen pyrolysis have achieved material reduction rates of up to 98.2% for EVA and 98.69% for PET. The authors further explain that experiments have shown stable gallium and arsenic recovery rates, with vacuum metallurgy providing a recovery efficiency of 93.48%. However, recycling can affect performance, indicating that the balance between sustainability and maintaining high efficiency is complex, yet essential, for future advancements.

## 4. Cadmium Telluride (CdTe)

Cadmium Telluride (CdTe) has emerged as a prominent semiconductor material in the field of thin-film solar cells. Characterized by its direct bandgap and high absorption coefficient, CdTe allows for the production of cells that are not only thinner but also more efficient in converting sunlight to electricity compared to traditional silicon-based cells [30]. This material has been at the center of significant research and development efforts, aiming to leverage its properties to create cost-effective, efficient, and scalable solar energy solutions. The use of CdTe in thin-film solar cells represents a pivotal advancement in photovoltaic technology, offering a promising pathway to achieving higher efficiency and lower production costs in solar energy systems [31].

The integration of CdTe into photovoltaic cells underscores the importance of material science in the ongoing evolution of solar energy technologies. As researchers continue to explore and refine the properties of semiconductor materials like CdTe, the potential for further enhancements in solar cell efficiency and the broader adoption of solar energy becomes increasingly tangible. This pursuit not only addresses the technical and economic challenges associated with solar energy but also contributes to the global effort to transition towards more sustainable and environmentally friendly energy sources [32].

Cadmium Telluride (CdTe) is distinguished by its high absorption coefficient, which enables it to absorb sunlight more efficiently than many other semiconductor materials [33]. This property allows CdTe solar cells to be manufactured with significantly thinner photovoltaic layers, without compromising their ability to capture solar energy. The thickness of a CdTe layer in a solar cell is typically less than 10 μm, which is considerably thinner than the silicon layers used in traditional photovoltaic cells, which can be over 200 μm thick [33]. This efficient use of material not only reduces the amount of raw material needed but also contributes to the overall efficiency of the solar cells by minimizing electron loss [34].

The production of CdTe solar cells is characterized by its cost-effectiveness, attributed to the simplicity of the manufacturing process and the low material usage [35]. The process for creating CdTe cells involves depositing a thin layer of CdTe material onto a glass or flexible substrate, which can be carried out at relatively low temperatures and with less energy compared to the production of silicon cells. This streamlined manufacturing process, coupled with the reduced material requirements, results in lower production costs, making CdTe solar cells a more affordable option for large-scale deployment [35]. The economic advantages of CdTe technology have been instrumental in its adoption and growth within the solar energy market [36].

The energy payback time (EPT) of CdTe solar cells is among the shortest for photovoltaic technologies [37]. EPT refers to the amount of time that a solar cell takes to generate the amount of energy that was used to produce it. Studies [37,38] have shown that CdTe solar cells have an EPT of less than one year, which is significantly lower than that of many silicon-based solar cells. This short EPT underscores the environmental friendliness of CdTe solar cells, as they quickly offset the energy consumed during their production, leading to a net positive energy generation over their lifespan [38].

CdTe solar cells exhibit superior performance across a wide range of temperatures and under low-light conditions [39]. Unlike some other photovoltaic materials, whose efficiency significantly drops at high temperatures, CdTe solar cells maintain a relatively stable output even in hot climates. Additionally, CdTe cells are effective at converting diffuse sunlight into electricity, which makes them particularly suitable for regions with frequent cloud cover or haze. This versatility enhances the applicability of CdTe solar cells in diverse geographical locations, contributing to their growing popularity in the global solar energy market [39].

One of the primary concerns associated with CdTe solar cells is the toxicity of cadmium, a heavy metal that is used in their production. Cadmium is recognized for its potential to harm human health and the environment if not properly managed [40]. The use of cadmium in consumer products, including photovoltaic cells, has raised questions about the long-term implications of CdTe solar cell disposal and the risk of cadmium release into the environment. However, industry practices have evolved to include rigorous safety standards and recycling programs aimed at mitigating these risks. Research into encapsulation and recycling technologies continues to minimize the environmental impact of cadmium in CdTe solar cells [40].

The limited availability of tellurium, a key component in CdTe solar cells, poses a challenge to the scalability of this technology [41]. Tellurium is one of the least abundant elements in the Earth’s crust, and its supply is predominantly derived as a byproduct of copper refining [41]. The scarcity of tellurium could limit the potential for significant expansion of CdTe solar cell production, prompting research into more abundant alternative materials or more efficient recycling methods to ensure the sustainable growth of CdTe photovoltaics [41].

The recycling and disposal of CdTe solar cells present challenges due to the need for specialized processes to safely handle and recover materials, particularly cadmium. While recycling programs have been developed to address these challenges, ensuring the safe and efficient recycling of CdTe solar cells at the end of their lifecycle is critical to minimizing their environmental impact [42]. Advances in recycling technologies and strategies are essential to support the sustainable deployment of CdTe solar cells, with ongoing research focused on improving the efficiency and accessibility of recycling processes [42].

Recent breakthroughs in the efficiency of Cadmium Telluride (CdTe) solar cells have been significant, with researchers achieving record cell efficiencies through advancements in junction quality and back contact improvements [43]. Innovations such as the development of high-resistivity transparent layers and the optimization of the CdTe/CdS junction have led to enhanced light absorption and reduced recombination losses, pushing CdTe cell efficiencies closer to their theoretical limits [43]. Additionally, the introduction of new back contact materials has improved the collection of carriers, further increasing the efficiency of CdTe solar cells [44]. These advancements not only enhance the performance of CdTe solar cells but also contribute to reducing the cost per watt of solar electricity, making solar energy more competitive with traditional energy sources. Alfadhili and group [45] explored the impact of methylammonium iodide treatment on developing a Te layer atop CdTe and ZnTe films for creating back contacts in CdTe solar devices. The authors developed eight different back contact designs to assess whether Te or ZnTe would enhance the device’s performance. Alongside performance metrics, the group measured barrier heights to better understand the back contact energetics. Their findings showed that incorporating Te or ZnTe as back contacts significantly improves VOC and device efficiency by optimizing band alignment.

Research into alternative materials and doping strategies aims to reduce the toxicity and improve the efficiency of CdTe solar cells. Efforts to replace or reduce the use of cadmium with less toxic materials without compromising cell performance are underway. For instance, the exploration of alternative buffer layers to replace the standard cadmium sulfide (CdS) layer with materials that have similar electronic properties but a lower environmental impact is a focus of current research [46]. Doping strategies involving the introduction of specific elements into the CdTe layer have been shown to enhance the material’s electrical properties, leading to improved cell efficiencies [47]. These advancements in material science not only address environmental concerns but also open new pathways for the development of high-efficiency, low-impact solar cells. Al-Kuhaili et al. [48] doped CdTe To overcome these concerns; samarium, a rare-earth metal known for its superior conductivity and high valence, was used for extrinsic doping. This study involved depositing samarium-doped CdTe thin films, with samarium concentrations ranging from 0 to 6.2 at%. The post-doping results showed a remarkable transformation in the films’ photoelectric properties, including a switch from p-type to n-type, an eightfold decrease in resistivity to 7.6 × 10^−2^ Ω⋅cm, successful ohmic contact formation, and a reduced optical bandgap that improved the solar absorption.

Innovations in manufacturing processes have played a crucial role in reducing the costs and improving the scalability of CdTe solar cell production. Advances in deposition techniques, such as close-spaced sublimation (CSS) and vapor transport deposition (VTD), have improved the uniformity and quality of CdTe layers while reducing production times and material waste [49]. The development of scalable manufacturing solutions, including roll-to-roll processing for flexible solar cells, has the potential to significantly lower production costs and expand the applicability of CdTe technology to a wider range of surfaces and applications [50]. These manufacturing innovations not only enhance the economic viability of CdTe solar cells but also contribute to the sustainability of solar energy as a scalable renewable resource. Siegler and group [51] explored the hurdles impacting the development of perovskite–CdTe tandem photovoltaics (PVs) and presented future directions for this structure. The authors crafted PVs with a wide-bandgap CH_3_NH_3_PbBr_3_ (MAPBr) as the top absorber layer, noting reasonable efficiency but encountering significant optical haze that restricted the CdTe layer’s light absorption and the tandem cell’s performance. Utilizing SCAPS software, the researchers simulated a four-terminal (4T) MAPBr-CdTe tandem setup, assessing the permissible haze level in the perovskite layer to enhance the efficiency compared with single-junction CdTe cells. 

The ongoing challenge of increasing the conversion efficiency of CdTe solar cells to compete with silicon-based cells remains a key focus of research and development. While recent advancements have significantly improved CdTe cell efficiencies, reaching and surpassing the efficiency levels of silicon-based solar cells requires continuous innovation in material properties, cell design, and manufacturing processes [52]. Efforts to understand and mitigate efficiency losses due to defects, grain boundaries, and interface recombination are critical to further advancements in CdTe solar cell technology [53].

CdTe solar cells face market challenges, including the public perception of cadmium’s toxicity and competition with other types of solar cells [54]. Addressing these challenges requires ongoing efforts to educate the public and stakeholders about the safety measures and environmental benefits of CdTe technology, as well as the competitive advantages of CdTe solar cells in terms of cost, efficiency, and versatility [54]. Overcoming market adoption barriers is essential to fully realizing the potential of CdTe solar cells in the global renewable energy landscape.

## 5. Copper Indium Gallium Selenide (CIGS)

Copper indium gallium selenide (CIGS) stands out within the thin-film category for its exceptional photovoltaic properties and potential for high-efficiency solar cells [55]. CIGS is a tetrahedrally bonded semiconductor with a chalcopyrite structure, which is key to its desirable photovoltaic characteristics. The material’s composition can be finely tuned to optimize its absorption spectrum, making it highly efficient at converting sunlight into electrical energy. This adaptability, combined with the material’s ability to be deposited on flexible substrates, positions CIGS solar cells as a versatile solution for a wide range of solar energy applications [56].

CIGS solar cells are distinguished by their high absorption coefficient and direct bandgap, which enable them to efficiently absorb sunlight and convert it into electricity [57]. The direct bandgap of CIGS can be adjusted to between 1.0 and 1.7 eV, allowing for optimal absorption of the solar spectrum. This tunability, combined with the material’s high absorption coefficient, means that CIGS solar cells can achieve high conversion efficiencies with significantly thinner absorber layers than those that are required for silicon-based cells. Recent advancements in CIGS technology have led to laboratory-scale cell efficiencies exceeding 22%, demonstrating the material’s potential for high-performance solar cells [57].

One of the most compelling advantages of CIGS solar cells is their compatibility with flexible substrates [58]. Unlike rigid silicon wafers, CIGS can be deposited on lightweight, flexible materials such as plastic, metal foils, or flexible glass. This flexibility opens up new applications for solar cells, including portable power sources, wearable electronics, and innovative building-integrated photovoltaic (BIPV) solutions. The ability to conform to different shapes and surfaces without compromising efficiency or performance makes CIGS an attractive option for integrating solar energy into everyday objects and structures [58].

CIGS solar cells also offer aesthetic advantages, with their uniform surface and the ability to customize their appearance through various deposition techniques [59]. This makes CIGS particularly suitable for building-integrated photovoltaics (BIPVs), where solar cells can be seamlessly integrated into architectural elements such as facades, roofing materials, and windows [59]. The potential for color customization, without significant loss in efficiency, allows for architectural designs that incorporate renewable energy solutions without compromising aesthetic values [59].

Recent achievements in CIGS cells’ efficiency have been remarkable, with laboratory records approaching or surpassing 23% [60]. These advancements are attributed to improvements in the cell structure, interface engineering, and the optimization of the absorber layer’s chemical composition [60]. For instance, the ZSW Laboratory in Germany reported a CIGS cell efficiency of 23.35%, setting a new world record for thin-film solar cell efficiency [60]. This milestone underscores the potential of CIGS technology to compete with traditional silicon-based solar cells in terms of efficiency. Jost and group [61] developed a monolithic perovskite/CIGS tandem solar cell achieving a certified 24.2% power conversion efficiency (PCE). Despite observing a photocurrent mismatch between subcells, the optimal device configuration was determined through optical simulations. The optimization suggested a theoretical PCE of 32% with a short-circuit current density of 19.9 mA cm^−2^. The energy yield assessment identified the CIGS temperature coefficient as −0.38% K^−1^, superior to the perovskite’s −0.22% K^−1^, indicating a better field performance at high temperatures. The tandem device significantly surpasses single-junction cells, with an over 50% energy output enhancement for CIGS, approaching the achievement of 30% PCE.

Advancements in non-vacuum deposition techniques, such as solution processing and printing, promise to lower production costs and increase the scalability of CIGS solar cell manufacturing [62]. These methods offer a more straightforward, energy-efficient, and potentially lower-cost alternative to traditional vacuum-based deposition techniques. For example, researchers have developed a non-vacuum electroplating method for CIGS layer deposition, demonstrating comparable efficiencies to vacuum-deposited cells but at potentially lower costs and with simpler equipment [62]. Goncalves et al. [62] integrated printing, coating, and chemical bath deposition to create solution-processed photovoltaic systems. Utilizing eco-friendly inks based on water and ethanol, the process involved screen-printing the photoabsorber onto glass coated with fluorine-doped tin oxide, followed by selenization, cadmium sulfide buffer deposition, and sputtering of intrinsic zinc oxide and aluminum-doped zinc oxide layers, achieving a maximum 6.6% efficiency—a record for screen-printed Cu(In,Ga)Se_2_ cells. A fully non-vacuum-processed device using spray-coated zinc oxide and tin-doped indium oxide layers reached 2.2% efficiency, marking significant progress in developing sustainable, efficient Cu(In,Ga)Se_2_ solar cells.

Research into alternative materials for the buffer and window layers aims to reduce costs and improve cell performance. Innovations include replacing the traditional cadmium sulfide (CdS) buffer layer with zinc magnesium oxide (Zn(Mg)O), which has been shown to enhance the cell’s voltage output and overall efficiency [63]. Additionally, the use of alternative window materials, such as zinc oxysulfide (Zn(O,S)), has been explored to improve the spectral response of CIGS cells [43]. He and group [64] explored the band structure of indium-doped Zn_1−x_Mg_x_O (ZMO:In) through first-principle calculations and SCAPS program simulation for ZMO:In/CdTe devices. The findings revealed an increase in ZMO’s band gap with Mg doping, which decreases upon In addition due to a lower conduction band. ZMO/In-based cells outperformed CdS-based ones, with a peak efficiency of 19.63% at a 0.0625 Mg concentration, benefiting from a wider band gap and a 0.23 eV conduction band offset. The optimal ZMO:In thickness for enhanced device performance was identified as 70–100 nm, providing a theoretical foundation for high-efficiency CdTe solar cell development.

The high cost of materials and challenges in scaling up production while maintaining high efficiency remain significant hurdles for CIGS technology [65]. The precision that is required in the deposition processes and the need for high-purity materials contribute to the overall cost of CIGS solar cells. Efforts to streamline manufacturing processes and reduce material costs are crucial for making CIGS a more competitive option in the solar market [65].

Concerns regarding the availability of indium and gallium, critical materials for CIGS production, persist [66]. Both elements are relatively rare and have competing uses in other technologies, which could limit the scalability of CIGS solar cells. Research into recycling these materials from end-of-life products and exploring alternative, more abundant materials for CIGS cells is ongoing [66,67]. 

Improving the long-term stability and durability of CIGS solar cells under various environmental conditions is an area of active research. While CIGS cells have demonstrated good operational stability, ensuring their performance over the typical 25-year lifespan of solar panels requires further advancements. Studies focusing on encapsulation techniques and the mitigation of potential degradation mechanisms are critical to enhancing the longevity of CIGS solar cells [68,69]. Zhang and group [69] addressed the urgent need for an effective encapsulation method for flexible CIGS to facilitate its market introduction. The authors presented an approach to encapsulate module-level (10 × 10 cm^2^) CIGS/glass solar cells using a thin Al_2_O_3_ barrier layer applied through atomic layer deposition (ALD). The results showed that a 10 nm thick ALD-Al_2_O_3_ layer was adequate for shielding the Al:ZnO (AZO) window layer from electrical degradation during damp heat tests (DHTs) and was similarly efficient in encapsulating 10 × 10 cm^2^ CIGS/glass mini-modules by blocking moisture. Mini-modules with the ALD-Al_2_O_3_ encapsulation maintained approximately 80% and 72% of their original efficiency after 1000 and 2000 h of DHTs, respectively, compared to unencapsulated modules, which fell to 67% (after 1000 h DHT) and 22% (after 2000 h DHT) of their initial efficiency. The authors further explained that the ALD-Al_2_O_3_ layer contributed to reduced electrical degradation in the AZO window layer and P3 interconnections, as well as fewer shunting paths, resulting in a smaller fill factor (FF) reduction in the encapsulated CIGS mini-modules. 

The potential for new alloys and doping strategies to enhance the efficiency and stability of CIGS solar cells is vast. Research is focused on identifying optimal compositions that can provide better bandgap tuning, improved absorption properties, and enhanced resistance to environmental degradation. The development of novel dopants and alloying elements could lead to significant improvements in cell performance and longevity [70,71].

The development of more cost-effective and scalable manufacturing processes, such as roll-to-roll printing and sputtering, is crucial for the widespread adoption of CIGS technology. These processes could enable the mass production of flexible, lightweight CIGS solar panels at lower costs, making solar energy more accessible and versatile. Innovations in manufacturing technology are expected to play a key role in the future growth of the CIGS market [72]. 

The expanding applications of CIGS solar cells, from traditional solar panels to innovative uses in consumer electronics, transportation, and urban infrastructure, highlight the versatility of this technology. The development of flexible, thin-film CIGS solar cells opens up new possibilities for integrating photovoltaic technology into everyday objects, buildings, and vehicles, contributing to the creation of more sustainable and energy-efficient environments [73].

## 6. Organic Photovoltaic Cells

Organic photovoltaic cells (OPVs) are a pivotal innovation in solar technology, distinguished by their utilization of carbon-based materials. These materials, including polymers and small molecules, are primarily organic semiconductors, setting OPVs apart from traditional inorganic solar cells [74]. The unique properties of these materials, such as their flexibility, semitransparency, and ability to be processed at low temperatures, make them highly adaptable for diverse applications, extending beyond the conventional scope of solar panels [74].

The journey of OPVs began with the discovery of the photovoltaic effect in organic materials. The initial breakthrough in OPVs was marked by the development of the “Tang cell” in 1986, a two-layer device comprising copper phthalocyanine and perylene diimide [75]. This discovery laid the foundation for the evolution of OPVs, leading to the concept of bulk hetero-junction (BHJ) solar cells in the early 1990s [75]. BHJ cells, composed of a blend of donor and acceptor materials, typically involve a combination of a polymer or small molecule donor with a fullerene-based acceptor. This structure facilitates efficient charge separation and transport, which are crucial for high photovoltaic performance [75].

A significant aspect of OPV research focuses on the synthesis and development of novel organic semiconducting materials. These materials are designed to optimize the light absorption, charge transport, and overall device efficiency [76]. Innovations in material science have led to the creation of various photovoltaic polymers, each offering distinct advantages in terms of absorption spectrum, molecular ordering, and electronic properties. For instance, the incorporation of carbon nanotubes and other nanostructured materials into the active layer of OPVs has been explored to enhance charge transport and improve the overall cell efficiency [76]. These advancements aim to address the inherent limitations of organic materials, such as their relatively narrow absorption spectra and less efficient charge carrier mobility compared to inorganic materials [76].

Despite the progress, OPVs face challenges, particularly in terms of efficiency and stability. The efficiency of OPVs, although improving rapidly, still falls short of their inorganic counterparts [77]. Stability is another critical issue, as organic materials are prone to degradation under environmental stressors like UV radiation, oxygen, and moisture. Addressing these challenges involves not only material innovation but also advancements in device engineering and encapsulation techniques. The ongoing research in this field is driven by the potential of OPVs to offer a low-cost, environmentally friendly, and versatile alternative to traditional solar technologies [77].

### 6.1. Advantages: Flexibility and Low-Cost Production Potential

The flexibility of OPVs is a standout feature, primarily due to the organic materials that are used in their construction. This flexibility enables OPVs to be integrated into a variety of applications where traditional, rigid solar cells are impractical [78]. For example, a study [78] demonstrated the potential of OPVs in indoor settings, showcasing their adaptability to different light conditions and environments.

The roll-to-roll manufacturing process is crucial in enhancing the low-cost production potential of OPVs. This technique, which involves printing photovoltaic materials onto flexible substrates, is less energy-intensive and more cost-effective compared to traditional silicon cell production methods [79]. Li and colleagues [79] focused on advancements in OPV cells. They discussed the development of a small molecule named DERHD7T, designed for improved solar absorption and film quality in OPV cells. This molecule achieved a record power conversion efficiency (PCE) of 6.1%, surpassing previous benchmarks for small molecule-based OPV devices. The study highlights the molecule’s synthesis, thermal stability, and performance characteristics, like high molar absorption, effective charge mobility, and device fabrication techniques. The research underscores the potential of small molecules in OPV technology, offering a promising alternative to polymer-based systems.

**Figure 4:**
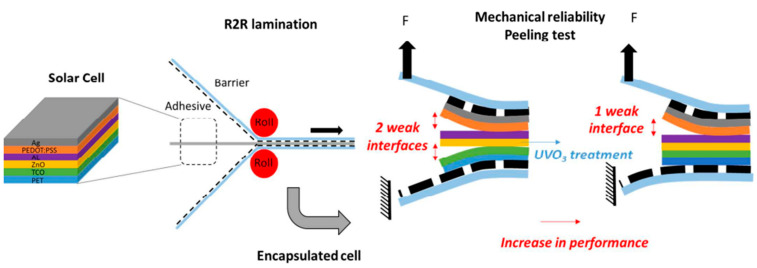
Process flow for enhancing the durability and performance of organic photovoltaic devices. The illustration shows a multilayered solar cell structure being encapsulated through roll-to-roll (R2R) lamination, identifying two weak interfaces that are prone to separation. A mechanical peeling test is used to assess the mechanical reliability. Subsequently, UV–ozone (UVO_3_) treatment is applied to one of the interfaces, resulting in improved adhesion, a reduction to one weak interface, and an overall increase in the solar cell’s performance. Reprinted with permission from ref. [80], copyright 2018, ACS Applied Materials and Interfaces.

The commercial viability of roll-to-roll manufactured OPVs hinges on effective encapsulation techniques. Encapsulation is essential for protecting organic materials from environmental degradation. Juillard et al. [80] investigated the impact of roll-to-roll flexible encapsulation on OPV devices, focusing on mitigating environmental degradation and enhancing device longevity. They conducted a comprehensive assessment of both the performance and mechanical reliability of encapsulated devices. Using a novel peeling technique, the authors analyzed the interfacial strengths within multilayered OPV devices on a flexible poly(ethylene terephthalate) substrate. This approach revealed significant weaknesses at two specific interfaces: between the active layer and the hole-transporting layer and between the transparent conducting electrode and the electron-transporting layer. To correct the weakness, the group explored various surface treatments, finding that optimized UV–ozone treatment significantly improved the adhesion of zinc oxide (ZnO) layers, as confirmed by IR spectroscopy and contact angle measurements (see Figure 4). The study concluded that enhancing interfacial adhesion not only improves device performance but also increases resilience to the stresses of roll-to-roll encapsulation.

Recent advancements have seen OPVs achieve significantly increased efficiencies, especially in indoor lighting conditions. Cui et al. [78] reported on the potential of OPV cells for indoor applications. OPV technology, characterized by its capacity for large-area, lightweight, and flexible solar panel production via low-cost roll-to-roll methods, has seen rapid improvements in its power conversion efficiency (PCE). Specifically, the group optimized OPV cells for indoor lighting conditions, achieving a top PCE of 22% with 1 cm^2^ cells under 1000 lux LED illumination. These cells also exhibited enhanced stability under continuous indoor light, underscoring the importance of developing wide-bandgap active materials that are tailored for indoor OPV applications, which could significantly elevate the photovoltaic performance.

### 6.2. Performance Metrics: Efficiency and Stability

Organic photovoltaic cells (OPVs) have seen significant advancements in terms of their power conversion efficiency (PCE) and stability, two critical performance metrics in solar technology. Recent developments in OPV technology have led to substantial improvements in the PCE. A notable example is the work by Wang et al. [81], where the authors explored the potential of OPV cells for indoor applications, addressing the challenge of energetic disorder under low illuminance. They demonstrated that concentrated indoor light mitigates energetic disorder, enhancing the open-circuit voltage and fill factor, with PB2:FCC-Cl (2,2′-((2Z,2′Z)-((12,13-bis(2-ethylhexyl)-3,9-diundecyl-12,13-dihydro-[1,2,5]thiadiazolo [3,4e]thieno[2″,3″:4′,5′]thieno[2′,3′:4,5]pyrrolo[3,2-g]thieno[2′,3′:4,5]thieno[3,2-b] indole-2,10-diyl)bis(methanylylidene)) bis(5,6-dicloro-3-oxo-2,3-dihydro-1H-indene-2,1-diylidene)) dimalononitrile)-based cells achieving a remarkable 33.0% PCE at 20,000 lux. Additionally, the group reported superior stability of OPV cells under such conditions, noting over 30,000 h of intrinsic lifetime for the PBDB-TF:Y6 ([(2,6-(4,8-bis(5- (2-ethylhexyl-3-fuoro)thiophen-2-yl)-benzo[1,2-b:4,5-b′]dithiophene))-alt-(5,5-(1′,3′-di-2-thienyl-5′,7′-bis(2-ethylhexyl)benzo [1′,2′-c:4′,5′-c′]dithiophene-4,8-dione))]: 2,2′-((2Z,2′Z)-((12,13-bis(2-ethylhexyl)-3,9-diundecyl-12,13-dihydro-[1,2,5]thiadiazolo[3,4e]thieno[2″,3″:4′,5′]thieno[2′,3′:4,5]pyrrolo[3,2-g]thieno[2′,3′:4,5]thieno[3,2-b] indole-2,10-diyl)bis(methanylylidene)) bis(5,6-difluoro-3-oxo-2,3-dihydro-1H-indene-2,1-diylidene)) dimalononitrile) system. The integration with optical waveguide concentrators suggests a pathway for low-cost manufacturing, underscoring the necessity of developing concentrated OPV cells for future indoor applications. Additionally, Ma et al. [82] presented a strategy for enhancing both the mechanical robustness and photovoltaic performance of all-polymer OPV cells, making them suitable for flexible wearable devices. The authors introduced a high-molecular-weight polymer donor, PBDB-TF,into a PBQx-TF:PY-IT(poly[4,8-bis(5-(2-ethylhexyl)thiophen-2-yl)benzo[1,2-b;4,5-b′]dithiophene-2,6-diyl-alt-(4-(2-ethylhexyl)-3-fluorothieno[3,4-b]thiophene-2-carboxylate-2-6-diyl)]) blend to improve the bulk hetero-junction morphology, resulting in more efficient charge transport and enhanced mechanical stress dissipation. This ternary blend film yields OPV cells with a maximum PCE of 18.2% and an impressive fill factor of 0.796, maintaining a PCE of 16.5% even under mechanical stress, offering a viable approach to fortify all-polymer OPV cells.

Despite these efficiency improvements, stability remains a major challenge for OPVs. The organic materials that are used in OPVs are often more susceptible to environmental degradation factors like oxygen and moisture. Wu and team [83] critically reviewed the recent research progress on the stability of high-performance OSCs, discussing factors limiting a device’s lifetime such as metastable morphology, air, irradiation, heat, and mechanical stresses. Their review emphasizes the need for ongoing research in material design and device engineering to enhance the stability of OPVs [83].

The efficiency of OPVs under standard solar conditions still lags behind that of conventional solar cells. However, their performance in indoor environments, as demonstrated by Wang et al. [81], suggests a niche where OPVs could be particularly effective. The unique spectral characteristics of indoor lighting compared to outdoor sunlight play a significant role in this context. The high tunability in the optical absorption and insensitivity to series resistance and active layer thickness make OPVs promising for indoor applications [81].

The bandgap of semiconducting polymers is a fundamental property that determines the range of solar spectrum that an organic solar cell (OSC) can effectively absorb [75]. The ideal bandgap for OSCs is typically in the range of 1.1 to 1.7 eV, which allows for optimal absorption of the solar spectrum while maintaining a high open-circuit voltage (Voc). Designing polymers with a specific bandgap requires precise control over the molecular structure, including the backbone conjugation length, the nature of side chains, and the introduction of donor or acceptor units within the polymer chain [76,80].

One challenge in specifying the bandgap is the trade-off between a wide absorption spectrum and the Voc. A narrower bandgap can increase the absorption of photons, particularly in the near-infrared region, but it can also lead to a reduction in the Voc, diminishing the overall power conversion efficiency (PCE) [71,74,75]. Conversely, a wider bandgap can enhance the Voc but at the expense of reducing the absorption range, leading to a decreased photocurrent. Molecular engineering strategies, such as the development of donor–acceptor (D-A) copolymers, have been employed to address this issue. These copolymers combine electron-rich (donor) and electron-deficient (acceptor) units, allowing for tunable bandgaps and improved absorption properties while maintaining satisfactory Voc levels [75].

Charge dynamics, encompassing charge generation, transport, and collection, are critical for the efficiency of OSCs. Upon absorption of sunlight, excitons (bound electron–hole pairs) are generated within the polymer matrix [77,83]. These excitons must dissociate into free charges and be transported to the electrodes without significant recombination losses.

The efficiency of exciton dissociation into free charges is highly dependent on the molecular architecture of the semiconducting polymers. The introduction of D-A interfaces within the polymer or between the polymer and a fullerene acceptor has been shown to facilitate exciton dissociation through the creation of charge transfer states [82]. However, designing polymers that promote efficient charge generation while minimizing energy losses remains challenging.

The mobility of charges (electrons and holes) within semiconducting polymers is another critical factor. A high charge mobility is essential to minimize recombination losses and ensure efficient charge collection at the electrodes [74]. The molecular ordering, crystallinity, and purity of the polymer significantly influence the charge mobility. Polymers with a high degree of crystallinity and ordered packing tend to exhibit higher charge mobilities. However, achieving such a high degree of order in solution-processed films is challenging. Molecular design strategies, including the introduction of side chains that promote self-organization and the synthesis of polymers with high molecular weights, have been explored to improve charge transport properties [74,76].

Efficient charge collection at the electrodes is crucial for maximizing the PCE of OSCs. The alignment of energy levels between the semiconducting polymer and the electrode materials plays a vital role in facilitating charge collection [79,80]. Misalignment can lead to significant energy barriers for charge extraction, increasing recombination losses. Molecular design strategies to tailor the end groups of polymers for better alignment with electrode materials are critical for optimizing the charge collection efficiency.

### 6.3. Technological Challenges and Prospects for Scalability

**Figure 5:**
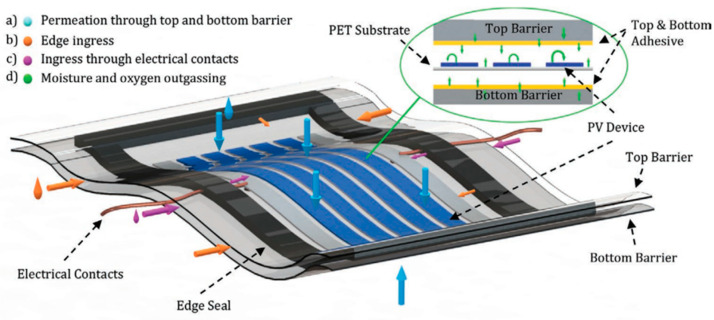
The illustration shows four distinct routes through which moisture and oxygen can penetrate flexible encapsulated PSC and OPV devices, leading to expedited degradation and diminished operational life. The permeation through top and bottom barrier (a) is represented by the light blue arrows and droplet, (b) edge ingress is represented by the light brown droplet and arrows, (c) ingress through electrical contact is represented by the violet droplet and arrows, and (d) moisture and oxygen outgassing is represented by the green droplet and arrows Reprinted with permission from ref. [84], copyright 2021, Advanced Energy Materials.

Scaling OPVs for widespread use involves overcoming several technological challenges, particularly concerning the inherent instability of organic materials and the complexities of large-scale production. The primary challenge in scaling OPVs is the inherent instability of organic materials. These materials can degrade under environmental stressors such as UV light, oxygen, and moisture. Sutherland et al. [84] argue that the development of flexible barrier encapsulation is essential, which requires materials with exceptional moisture resistance, high transparency, and durability against mechanical stress. Their review discusses these challenges in detail and examines the latest advancements in flexible encapsulation materials, suggesting directions for future research. Figure 5 shows four distinct routes through which moisture and oxygen can penetrate flexible encapsulated PSC and OPV devices.

**Figure 6:**
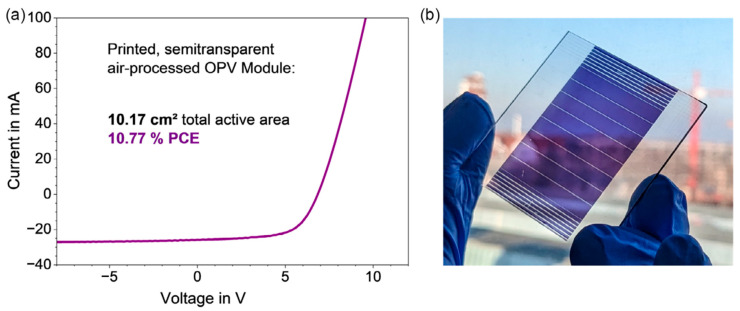
The image shows (**a**) the J-V curve and (**b**) a photograph of a semitransparent module that has been fully processed in solution and in air. This module, positioned on a glass/ITO substrate, consists of eight cells connected in series and covers a total active area of 10.17 cm^2^, including a back-reflector. Reprinted with permission from ref. [85], copyright 2023, Solar RRL.

Transitioning from small-area lab cells to large-scale, industrially viable OPV modules presents significant challenges. Wachsmuth and colleagues [85] explored the upscaling of OPVs from small-area lab cells to solution-processed modules that are compatible with industrial roll-to-roll (R2R) printing. This process involved meticulous material selection and optimization of each layer in the OPV stack, including the photoactive and charge-transporting layers, as well as the solution-processed top electrode. The authors also conducted long-term stability tests (thermal and light exposure) and successfully scaled up the device area by over 100 times. The result was a semitransparent OPV module with a 10.8% power conversion efficiency on a 10.2 cm^2^ area, meeting industrial R2R printing requirements, thus paving the way for large-scale production (see Figure 6).

Encapsulation plays a crucial role in enhancing the stability of OPVs, especially in the context of large-scale production. E. Destouesse et al. [86] successfully implemented both roll-to-roll (R2R) and sheet-to-sheet (S2S) methods to develop ITO-free OPV devices using the non-fullerene PBDB-T:ITIC material system. The fabrication involved R2R vacuum sputtering and S2S slot-die coating, all conducted under ambient conditions, yielding devices with a power conversion efficiency of 5.5%. The authors also investigated the relationship between various barrier films, including commercially available and sputtered inorganic coatings on ultra-clean PET, as well as the longevity of these OPV devices. The findings mark a significant advancement in the industrial-scale production of OPV devices.

The future of OPVs in large-scale applications hinges on addressing these challenges. Continued advancements in material stability, encapsulation techniques, and upscaling processes are crucial for the long-term viability and commercial success of OPVs. As these technological hurdles are overcome, OPVs hold the promise of becoming a key player in the renewable energy sector, offering a sustainable and cost-effective alternative to traditional solar technologies.

## 7. Perovskite Solar Cells

Perovskite solar cells (PSCs) have rapidly emerged as a promising photovoltaic technology, primarily due to their excellent optoelectronic properties and ease of fabrication [87]. A review by Yang et al. [85] focuses on the advancements of all-inorganic CsPbX_3_ perovskites, known for their excellent optoelectronic properties and stability, with applications in electronic devices. The power conversion efficiency of CsPbX_3_ perovskite solar cells has remarkably increased from 2.9% in 2015 to 21.0%. Emphasis is placed on optimizing the quality of perovskite films through crystallization kinetics modulation and defect suppression. The review covers fundamental aspects of CsPbX_3_ perovskites, strategies for crystallization modulation, and methods to enhance inorganic perovskite solar cell efficiency, while also discussing future development prospects.

Organic–inorganic hybrids are known for their broad absorption spectra, long recombination lifetimes, and impressive electron mobility [88]. Zhou et al. [89] delved into the progression of perovskite photovoltaic technology, highlighting its rapid development due to advances in understanding the thin-film microstructures of metal halide perovskites. The authors focused on three crucial microstructure types: grain boundaries, intragrain defects, and surfaces. Their impacts on optoelectronic properties, device efficiency, and stability were discussed, emphasizing the importance of tailored characterizations to understand these effects. They also examined the microstructures’ roles in degradation modes and presented examples where fundamental insights have led to state-of-the-art perovskite solar cells. The paper concludes with a call for further exploration of hidden microstructures and advanced characterizations to enhance our understanding of microstructure–property–performance relationships in solar cells.

The unique economic advantage of PSCs lies in their solution-processible fabrication, which is compatible with large-scale deposition techniques such as roll-to-roll processing, blade coating, or inkjet printing [90]. This adaptability potentially lowers solar cell fabrication costs significantly, making them a key player in the renewable energy sector. Additionally, the exploration of lead-free perovskite materials, such as antimony-based perovskites, is gaining traction due to their unique optoelectronic properties, conventional fabrication processes, low toxicity levels, and high stability values, as shown by Thomas [90]. Similarly, Chakraborty et al. [91] examined the potential of various lead (Pb)-free materials for use in hybrid halide perovskite solar cells (PSCs). Their work highlights the research efforts to find suitable lead alternatives in PSCs, with a focus on homovalent (Sn^2+^, Ge^2+^, Cu^2+^) and heterovalent (Sb^3+^, Bi^3+^, Ti^4+^) materials. Although these materials show promising physical properties to replace lead, their power conversion efficiencies (PCEs) are generally lower compared to their lead-based counterparts.

### 7.1. Halide Perovskites

Halide perovskites have emerged as a leading material in the field of photovoltaics due to their remarkable optoelectronic properties, which include high absorption coefficients, tunable bandgaps, and long carrier diffusion lengths [92]. These properties are intimately linked to their unique crystal structure and the dynamic behavior of their charge carriers, which together contribute to the high efficiencies observed in perovskite solar cells.

The crystal structure of halide perovskites is defined by the ABX₃ formula, where “A” is a monovalent cation such as methylammonium (MA), formamidinium (FA), or cesium (Cs); “B” is a divalent metal cation, typically lead (Pb) or tin (Sn); and “X” is a halide anion (Cl, Br, or I) [93]. This structure forms a three-dimensional network of BX₆ octahedra, with the A cations located in the interstitial spaces. The flexibility in the choice of A, B, and X components allows for the tuning of the material’s bandgap by altering the crystal lattice dimensions and the octahedral tilting, which is a critical aspect for optimizing solar cells’ performance [94].

The perovskite crystal structure is known for its tolerance to defects, which is partly due to the self-healing nature of the ionic lattice [95]. This tolerance contributes to the high defect tolerance of these materials, allowing them to maintain high charge carrier mobilities and lifetimes despite the presence of defects that would typically act as recombination centers in other semiconductor materials.

The optoelectronic properties are directly influenced by their crystal structure [96]. These materials exhibit direct bandgaps, which can be tuned from the ultraviolet to the near-infrared spectrum by varying the halide composition. This tunability enables the design of perovskite solar cells that can efficiently absorb a wide range of the solar spectrum [96].

Halide perovskites also possess high charge carrier mobilities and long diffusion lengths, which are essential for efficient charge collection in solar cells [97]. These characteristics are attributed to the strong absorption coefficients and the relatively low effective masses of electrons and holes, facilitating rapid and efficient charge separation and transport [97].

Another notable feature is the high photoluminescence quantum yield of halide perovskites, which is indicative of low non-radiative recombination rates. This property is beneficial for achieving high open-circuit voltages in solar cells, as it reflects the material’s ability to efficiently convert absorbed photons into electrical energy [98].

Despite their advantages, halide perovskites face challenges related to their stability under environmental conditions, such as moisture, oxygen, and light exposure [99]. The ionic nature of their crystal lattice makes them susceptible to degradation, which can lead to performance loss over time. Furthermore, the presence of lead in most high-performance perovskite solar cells raises environmental and health concerns, driving research towards lead-free alternatives [99].

### 7.2. Efficiency Gains and Fabrication Techniques

Perovskite solar cells (PSCs) have demonstrated remarkable progress in power conversion efficiencies (PCE), with recent reports indicating efficiencies reaching up to 26.1% [100]. This rapid improvement in PCE is attributed to advancements in fabrication techniques and material engineering. Pathak et al. [100] discuss the evolving technological requirements for effective energy production and conversion, with a focus on the rise of sustainable and renewable energy sources, particularly solar energy conversion via photovoltaic cells. The work highlights the significant increase in the power conversion efficiency of perovskite solar cells from 3% to 26.1% and the challenges in transitioning from laboratory PSCs to commercialization. Key topics include scalable fabrication processes for perovskite solar modules (PSMs), fabrication challenges, recent advancements in PSM stability, and future prospects for PSMs, providing insights into thin-film coating technologies and future development directions.

Innovative fabrication methods, such as the electro-hydrodynamic spraying route inspired by the “Marangoni flow”, have enabled the production of perovskite thin-film solar cells with superior current–voltage characteristics [101]. This method not only enhances efficiency but also reduces lead wastage, addressing environmental concerns associated with PSCs. Pourjafari et al. [101] focus on carbon-based, hole-conductor-free perovskite solar cells (C-PSCs), a promising candidate for commercial photovoltaic technology due to their high stability, ease of fabrication, and low cost. The authors explore various strategies to enhance charge separation, extraction, and transport in C-PSCs to improve power conversion efficiency. These include the use of novel or modified electron and hole transport materials and carbon electrodes. Their work also covers the working principles of printing techniques for C-PSC fabrication and discusses scalable deposition techniques for manufacturing perovskite solar modules.

**Figure 7:**
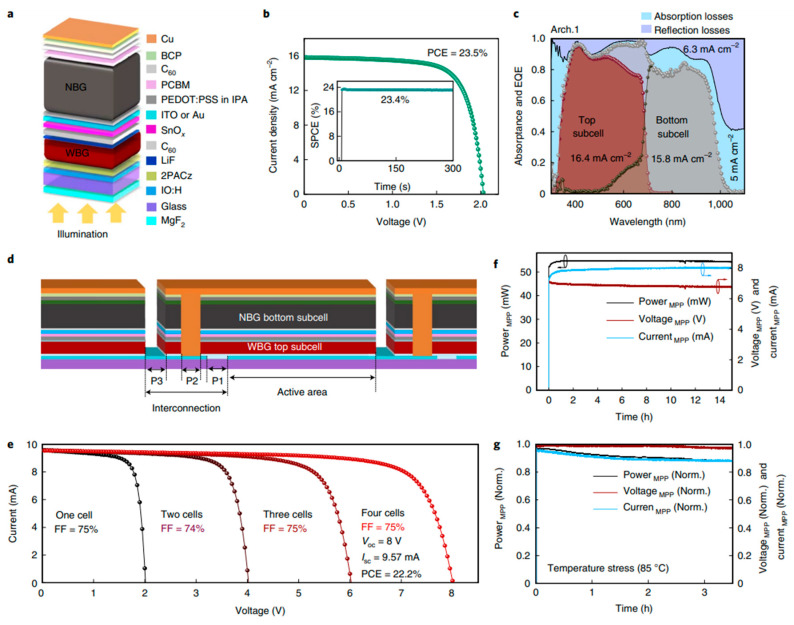
Development and efficacy of comprehensive perovskite-based tandem solar cells and modules are detailed. (**a**) outlines the structural design and composition of these tandem solar cells, highlighting the use of sputtered indium tin oxide layers (around 15 nm thick) and thinly spread Au films (about 1–2 nm thick) as the recombination layer. “NBG” and “WBG” refer to the narrow and wide bandgaps, respectively. (**b**) shows the current density–voltage (J–V) profile and efficiency of power conversion at the maximum power point over five minutes for top-performing tandem devices (see inset). (**c**) discusses the external quantum efficiency (EQE) of the upper and lower subcells, along with their combined effect (shown as a grey line) and the overall absorption, calculated as 1 minus the reflectance (depicted as a black line). This section also explains the light and dark blue areas, which indicate the parasitic absorption and reflection losses, respectively, and their impact on current density. (**d**) provides a diagrammatic representation of the interconnected two-terminal all-perovskite tandem solar module, not to scale, highlighting its active area and scribing lines. The color scheme for the module layers matches that of the tandem solar cells. I describes the J–V characteristics of individual tandem cell stripes within the module, including fill factors and the impact of progressively incorporating cell stripes into the analysis. (**f**) details the power, voltage, and current at the module’s maximum power point under continuous AM 1.5G lighting. (**g**) presents the normalized power, voltage, and current at the maximum power point when subjected to temperature stress at 85 °C in a nitrogen environment. The first three sections (**a**–**c**) pertain to tandem solar cells, while the latter sections (**d**–**g**) focus on tandem modules. Reprinted with permission from ref. [102], copyright 2022, Nature Energy.

Furthermore, the work by Nejand et al. [102] developed monolithic all-perovskite tandem photovoltaics, which combine the benefits of low-cost and high-efficiency solar energy harvesting that are inherent to all-thin-film technologies. The authors explained that until now, such tandem solar cells have been limited to lab-scale production using non-scalable techniques. Addressing this, their work introduced all-perovskite tandem modules that were fabricated through scalable methods like blade coating and vacuum deposition. These modules showcased power conversion efficiencies of up to 19.1% over a substantial aperture area of 12.25 cm^2^. They also maintained a high geometric fill factor of 94.7% and exhibited a stable power output (see Figure 7). When compared to their spin-coated tandem cells, which have an efficiency of 23.5% over a much smaller area, these scalable modules marked a significant step forward in making all-perovskite tandem photovoltaics more commercially viable. Electroluminescence imaging and laser-beam-induced current mapping techniques were employed to ensure uniform current collection across the module, minimizing losses in key areas like the open-circuit voltage and fill factor.

**Figure 8:**
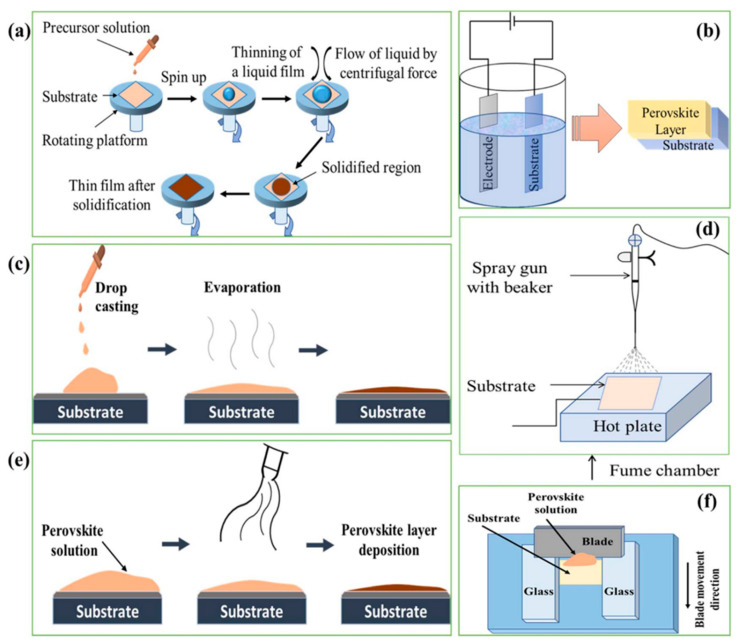
Fabrication methods for solution-based perovskite solar cells (PSCs) include (**a**) the spin-coating technique, (**b**) the method of electrochemical deposition, (**c**) the drop-casting approach, (**d**) spray coating procedure, (**e**) the blow-drying process, and (**f**) blade coating techniques. Reprinted with permission from ref. [87], copyright 2022, RSC Advances.

Chowdhury et al. [87] explored the effects of perovskite films, which show a concerning 20% performance degradation. The study emphasized the need for a deep understanding of the fabrication process’s impact on the device stability, considering it crucial for future progress. The authors provided a detailed examination of various fabrication methods, including spin-coating, doctor blade, sequential deposition, hybrid chemical vapor deposition, and layer-by-layer approaches (see Figure 8). The group also covered the evolution of PSC structures, transitioning from regular to inverted configurations, and shifts in material usage from organic to inorganic, highlighting innovations in perovskite materials. A key focus is the operational stability of PSCs, offering insights into extending their operational life and thus promoting their commercialization by overcoming the stability hurdle.

### 7.3. Comparative Analysis with Silicon and Organic Cells

Perovskite solar cells have emerged as a competitive alternative to traditional silicon-based solar cells, offering a unique blend of high efficiency and low-cost production potential. Hussain et al. [103] highlight that while silicon-based solar cells are approaching their efficiency limits, perovskite-based cells have demonstrated efficiencies of approximately 26%, surpassing many conventional silicon cells [103]. This remarkable efficiency, combined with the low-cost production techniques, similar to those used in organic photovoltaics, positions PSCs as a potential bridge between the high efficiency of silicon cells and the economic advantages of organic cells.

Giannouli [104] presents a comprehensive comparative assessment of third-generation photovoltaic technologies, including dye-sensitized solar cells (DSSCs), organic solar cells (OSCs), and PSCs, as alternatives to silicon solar cells. This study emphasizes the need for further research to improve the efficiency and stability of these devices while keeping production costs minimal. PSCs, in particular, are noted for their rapid development and potential for sustainable solar energy applications [104].

The work of Zhu et al. [105] reported a notable advancement in perovskite solar cell technology through the integration of two-dimensional perovskites within a three-dimensional framework, a method that traditionally enhances stability but has struggled with achieving high power conversion efficiencies. The authors’ approach breakthrough involved incorporating n-type, low-optical-gap conjugated organic molecules into this 2D:3D perovskite composite, resulting in ternary perovskite–organic composites. These composites showed extended absorption in the near-infrared region, improved film morphology, larger crystallinity, balanced charge transport, efficient photoinduced charge transfer, and reduced counter-ion movement. This approach has led to solar cells with PCEs over 23%, which are among the highest for perovskite solar cells with a p–i–n structure, alongside significantly enhanced stability and reduced photocurrent hysteresis. This study highlights the potential of ternary perovskite–organic composite thin films in developing high-performance perovskite solar cells, combining improved stability and efficiency.

**Figure 9:**
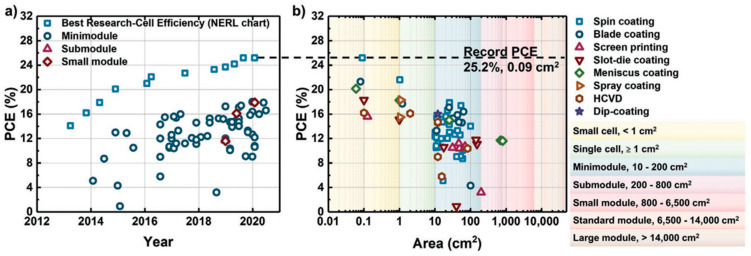
(**a**,**b**) Progression Trends in perovskite solar cells: (**a**) depicts the power conversion efficiency (PCE) over the years of various cell sizes; (**b**) shows the relationship between PCE and cell size for different fabrication methods. Reprinted with permission from ref. [106], copyright 2020, Advances Materials.

Lee et al. [106] presented an in-depth analysis of the challenges and current status of upscaling perovskite solar cells for commercialization, noting a significant efficiency gap between a large 804 cm^2^ perovskite module (17.9% efficiency) and a much smaller 0.09 cm^2^ cell (25.2% efficiency). To contextualize these findings, the authors explored the development and upscaling history of commercialized solar technologies, including silicon, copper indium gallium sulfur/selenide (CIGS), and CdTe, with module sizes reaching approximately 25,000 cm^2^ (see Figure 9). The study also examined other photovoltaic technologies such as GaAs, organic, dye-sensitized, and perovskite/silicon tandem solar cells, analyzing their operating mechanisms and development paths. The study allowed the group to draw parallels and contrasts in development strategies across different solar cell types, leading them to propose an optimal direction for the upscaling of perovskite solar cells. The authors concluded that lessons from the historical evolution of various solar technologies offer a fundamental understanding of the relative and absolute development stages of perovskite solar cells, providing a unique perspective that could guide the upscaling and advancement of this promising technology.

In terms of the market, the global photovoltaic (PV) market has witnessed exponential growth over the past decade, driven by the urgent need for renewable energy sources [82]. Within this broad landscape, silicon-based solar cells have traditionally dominated the market, benefiting from mature technology, high efficiency, and extensive industrial scaling. However, the landscape is gradually evolving with the advent of new materials and technologies, among which perovskite and organic photovoltaic (OPV) solar cells stand out due to their promising features. Despite their potential, perovskite and OPV technologies currently occupy a small fraction of the global PV market, a status reflecting both their nascent stage of development and the challenges that they face in terms of scalability, stability, and integration into the existing energy infrastructure [85,92].

Perovskite solar cells, in particular, have garnered significant attention for their remarkable efficiency improvements, achieving record-breaking efficiencies that rival and sometimes surpass those of conventional silicon cells within just a decade of research [95,96,97]. Their efficiency, combined with the low cost of materials and potential for simple manufacturing processes, positions them as a strong candidate for future market expansion. As of April 2023, perovskite solar cells were transitioning from laboratory-scale research to pilot production and commercial trials [101]. However, they still represented a minor share of the global PV market, primarily due to lingering concerns over their long-term stability, the toxicity of lead-based perovskites, and the need for robust encapsulation techniques to ensure operational longevity.

Organic photovoltaics, offering unique advantages such as flexibility, transparency, and the potential for low-cost roll-to-roll production, have also made significant strides [82,84]. OPVs have carved out niche applications where their unique properties offer distinct benefits, such as in building-integrated photovoltaics (BIPVs) and portable electronic devices. Despite these advancements and a steady improvement in efficiencies, reaching over 10% for some OPV systems, their market share remains small compared to conventional PV technologies [85]. The primary challenges hindering OPV market penetration include their lower efficiency relative to silicon and perovskite solar cells, issues with operational stability, and the scaling up of production processes. As the technology matures and solutions to these challenges are developed, it is anticipated that OPVs will gain a larger foothold in the market, especially in applications where their unique attributes can be fully leveraged.

### 7.4. Stability and Environmental Impact Considerations

Perovskite solar cells face significant challenges related to their stability and environmental impact, which are critical barriers to their commercialization. Kymakis [107] emphasizes that the long-term stability of PSCs under ambient operating conditions, particularly against environmental hazards such as heat and moisture, is a major hurdle. The degradation of PSCs under prolonged illumination and chemical decomposition due to the presence of water, thermal stress, UV radiation, and electrochemical reactions at the interfaces are key issues that need addressing. The best recorded operating lifetime of PSCs is 1000 h at maximum power and 60 °C for mesoscopic solar cells, highlighting the importance of interface engineering in improving stability [107].

Dou and Chen [108] discuss the degradation mechanisms in perovskite solar cells and provide a thorough review of interfacial engineering, with a particular focus on its effects on flexible perovskite solar cells. Based on recent research progress, the authors examine the current challenges and future directions, aiming to contribute to the advancement and commercialization of flexible perovskite solar cells. Their work offers valuable insights and perspectives, underscoring the importance of interface engineering in enhancing the performance of flexible photovoltaic technologies.

**Figure 10:**
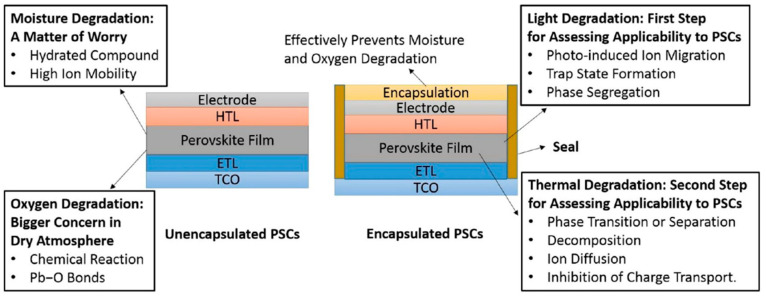
Comprehensive overview of degradation mechanisms in perovskite absorbers due to external environmental influences. Reprinted with permission from ref. [109], copyright 2021, ACS Chemistry of Materials.

Chi and Banerjee [109] reported on the advancements and ongoing challenges in metal halide perovskite solar cells, which are nearing their theoretical efficiency limits thanks to global research efforts. The authors explained that the current challenge lies in developing devices that not only achieve these efficiencies but also demonstrate sufficient stability and minimal degradation for practical applications. They identified that degradation in these cells is significantly influenced by external factors such as moisture, oxygen, light, and heat (see Figure 10). While encapsulation effectively counters moisture- and oxygen-induced degradation, mitigating light and heat degradation requires enhancing the materials and interfaces of the cells. Their study elaborated on the degradation mechanisms due to light and heat in each major layer of the device and discussed strategies for degradation reduction and stability enhancement. These strategies involve compositional and interfacial engineering approaches, such as site-based substitution in the perovskite lattice, doping in charge transport layers, and various passivation methods using materials like small molecules, polymers, and perovskite quantum dots. Their findings provide crucial insights into the suppression of degradation and the enhancement of stability in perovskite solar cells, guiding the design of efficient and durable solar energy devices.

**Figure 11:**
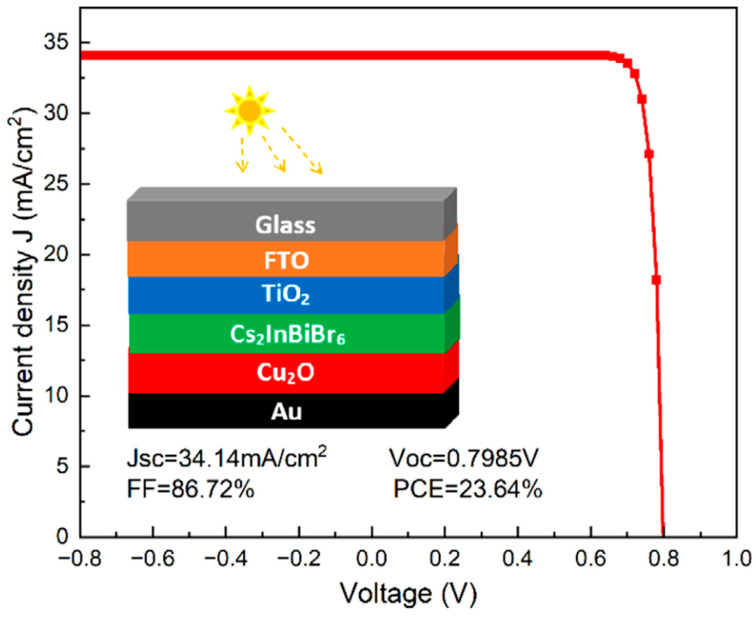
Comprehensive overview of degradation mechanisms in perovskite absorbers due to external environmental influences. Reprinted with permission from ref. [110], copyright 2023, Advanced Theory and Simulations.

Meng et al. [110] present a significant advancement in third-generation photovoltaic technology, focusing on the development of lead-free perovskite solar cells. Addressing environmental and health concerns associated with lead (Pb), the authors introduce a novel lead-free double perovskite material, Cs_2_InBiBr_6_, which is notable for its small direct bandgap of 1.27 eV and excellent thermodynamic and mechanical stability. Their research involves using a solar cell capacitance simulator to analyze a cell structure comprising FTO, ETL, Cs_2_InBiBr_6_, HTL, and Au, aiming to optimize the efficiency by selecting appropriate hole transport and electron transmission materials. They also explore the effects of absorber layer thickness, doping densities, and total defect density on cell performance. Employing advanced characterization methods like Mott–Schottky analysis, the group investigated the impact of interfaces on device functionality. Their findings revealed that a solar cell configuration of FTO/TiO_2_/Cs_2_InBiBr_6_/Cu_2_O/Au achieves an impressive PCE of 23.64% (see Figure 11), demonstrating the significant potential of Cs_2_InBiBr_6_ as a lead-free double perovskite solar cell absorber layer. This breakthrough indicates a promising future for environmentally sustainable and efficient perovskite solar cells.

Table 1 provides a comprehensive comparison of three key solar cell technologies: silicon-based, organic, and perovskite. It systematically outlines the advantages, disadvantages, and recent advancements of each type, offering insights into their market positions, technological strengths, and challenges. The advantages section emphasizes factors like efficiency, cost, and application versatility, while the disadvantages highlight environmental, stability, and manufacturing concerns. The recent accomplishments section focus on groundbreaking research and technological improvements.

## 8. Cross-Material Analysis

### 8.1. Efficiency and Stability of Photovoltaic Materials

The effective deployment of photovoltaic materials in commercial systems is extensively influenced by their conversion efficiency, which is bounded by the theoretical Shockley–Queisser (S-Q) limit [111]. This limit is a key determinant in understanding the maximum potential efficiency of photovoltaic cells based on their semiconductor material’s inherent properties. The efficiency of these materials is further impacted by their stability over time and the intricacies involved in their manufacturing. The solar spectrum, covering a vast range of wavelengths, interacts differently with various photovoltaic materials, influencing their ability to absorb light and convert it into electricity.

Materials like silicon have specific energy thresholds for photon absorption, dictating their efficiency in energy conversion. For instance, in silicon-based cells, photons with energy below 1.1 eV fail to induce excitation. Any excess photon energy, rather than contributing to the electrical output, increases the temperature of the cells, negatively impacting their efficiency. This absorption spectrum varies widely among different materials, each with its theoretical efficiency limit, often below the maximum potential outlined by mono-crystalline silicon [112].

Moreover, the efficiency of photovoltaic cells is not solely dependent on the material’s inherent properties. External factors such as reflections from the cell surface, the electrical resistance within the cell, contamination of the active components, and the presence of crystal defects critically affect the cell’s performance [113]. These factors collectively contribute to losses in efficiency, underlining the complexity of optimizing solar energy conversion [113].

In addition to these material and design-related factors, practical aspects such as the alignment of the solar panels, the angle of the sun’s incidence, and the geographical location of the installation play significant roles [114]. These factors influence the operational temperature, the amount of sunlight that is received per day, and the heat levels within the system, all of which have a direct impact on the efficiency of the photovoltaic systems.

The effectiveness of crystalline silicon solar cells, for example, is significantly influenced by the absorption factor, which is a measure of the solar irradiance that the cells can capture. This factor is critical for regulating the temperature of the cells and can be experimentally determined through reflection and transmission studies. Innovations in the texture of crystalline silicon wafers have been shown to enhance this absorption factor, thereby reducing reflective losses and potentially increasing the efficiency of the cells [115].

In the realm of bifacial crystalline silicon photovoltaic cells, there is a growing interest due to their potential for higher energy yields [116]. These cells are distinguished from monofacial cells by their ability to absorb light from both sides, which is facilitated by advanced performance metrics and sophisticated simulation models [116].

Recent advancements in silicon hetero-junction solar cells and the development of carrier-selective contacts have shown promising results in enhancing the efficiency of photovoltaic cells [117]. Furthermore, research into hybrid polymer semiconductor materials has shed light on their photon absorption and exciton generation capabilities. While these materials currently exhibit lower efficiencies compared to traditional semiconductor devices, they represent a significant area of research and development in the quest for more efficient photovoltaic solutions [117].

**Figure 12:**
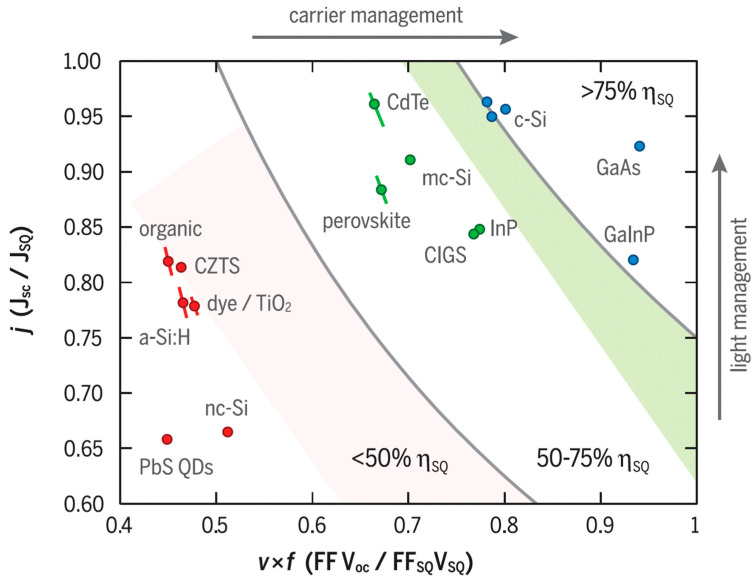
Fraction of the S-Q theoretical maximum for voltage and current attained by the different photovoltaic materials. The arrows displayed on the top and right axes of the graph represent the enhancement of cell efficiency through better light management and augmented charge carrier collection. The term “η_SQ_” is used to denote the maximum efficiency achievable as per the S-Q model. Reprinted with permission from ref. [119], copyright 2016, Science.

In solar cells, the inability to absorb all incident light and collect all generated carriers results in a lower short-circuit current than the maximum achievable current for a given band gap (Eg) [118]. The open-circuit voltage is also reduced from the ideal S-Q limit due to various recombination phenomena, as well as defects in the bulk, interface, and surface of the cells [57]. Additional losses from resistance, contact issues, and other nonideal factors further decrease the fill factor, which is a measure of the cell’s operational efficiency. Consequently, these factors result in practical efficiencies that are substantially lower than the theoretical maximum that is defined by the S-Q limit for a given band gap. In this regard, Polman et al. [119] conducted a comprehensive analysis of various photovoltaic materials, as illustrated in their study (see Figure 12). They assessed two critical parameters for each material: (i) the current ratio j = J_sc_/J_SQ_, reflecting the effectiveness of light coupling, absorption, and entrapment in the cell’s active layers, and its dependence on the carrier’s collection efficiency; and (ii) the voltage ratio v = V_oc_/V_SQ_, associated predominantly with carrier recombination in the bulk, surfaces, and interfaces [58]. The combination of the voltage ratio v and the fill factor ratio f = FF/FF_SQ_ serves to delineate the overall electrical constraints of a cell. Figure 12 displays the proportion of the S-Q detailed balance limit achieved for voltage and current by the analyzed materials, with lines around certain data points indicating the variability in band gaps used in the S-Q calculations, reflective of uncertainties in the band gap of the best-performing cell [119].

Conversion efficiency data, while crucial, do not fully capture the durability or longevity of solar cells in delivering a maximum output or maintaining their photovoltaic properties after production [120]. First-generation solar cells, notably those based on silicon, have shown remarkable durability, with some units still being operational decades after installation. This longevity is contrasted with the challenges that are faced by third-generation solar cells, particularly in maintaining internal stability over extended periods [121]. Technological advancements in the 21st century have brought significant developments in organic and hybrid photovoltaic research. These newer types of solar cells offer advantages such as lower production costs and a reduced environmental impact. However, their market adoption is hindered by their vulnerability to environmental factors like atmospheric conditions and biological agents, which can lead to rapid degradation [121]. The degradation rates for silicon and thin-film solar cells have been extensively documented since their commercial inception over fifty years ago. Most manufacturers of photovoltaic modules offer warranties of 25 to 30 years, aligning with an expected power drop of less than 20% during that period [121]. Recent models of solar cells, manufactured post-2000, show varied but generally lower degradation rates across different types of silicon cells. Some companies have reported some of the lowest degradation rates in the industry, achieved through innovative material choices [122]. Despite these advancements, third-generation solar cells, particularly those based on organic materials, face significant challenges. They often struggle to achieve efficiencies above 10% and are prone to rapid decomposition under exposure to light, drastically reducing their operational lifespan. This presents a major limitation for the practical application of these materials in long-term solar energy solutions.

Recent advancements in solar technology have significantly enhanced the robustness and efficiency of materials that are used in solar photovoltaic systems, enabling them to withstand extreme weather conditions and temperatures in various environments. In particular, perovskite solar cells have emerged as a key area of innovation in terms of long-term stability. The integration of nano-scale metal–organic frameworks (MOFs), with their flexible structures and expansive surface areas, has proven vital in improving the stability and performance of perovskite cells [123]. To further advance these technologies, a comprehensive understanding of the processes of photodegradation and thermal degradation in hybrid perovskite cells is crucial. Additionally, employing interfacial engineering techniques with hydrophobic materials and exploring the 2D/3D design concept have been pivotal in enhancing the long-term stability of these photovoltaic materials [123]. These technological strides represent a significant departure from the early stages of solar technology, demonstrating the growing potential of solar photovoltaic systems to remain durable and efficient over prolonged periods.

### 8.2. Commercial Viability and Scalability

In the field of photovoltaic materials, every practical material that is used for the development of photovoltaic devices undergoes continuous processes of standardization and reformulation. This is crucial for enhancing their properties, characteristics, longevity, and practical viability. Improvement is a possibility for all materials, and this section will explore such advancements, particularly focusing on materials like silicon, organic materials, and perovskite. These materials are at the forefront of research and development in the photovoltaic field, with ongoing efforts to optimize their performance and applicability in solar energy technologies.

Crystalline silicon, accounting for approximately 90% of the global photovoltaic market, has experienced steady growth over the years [124]. Despite this, alternatives to improve their efficiency and reduce associated costs have been explored. Recently, advancements in efficiency, manufacturing processes, material savings, and economies of scale have significantly reduced production costs, with reductions nearing 75%. However, these improvements, particularly in cost reduction, are not sufficient to meet climate targets set by the Panel on Climate Change for using photovoltaic technology [125]. Thin silicon wafers emerged as a cost reduction strategy, initially lacking market momentum. Consequently, reducing silicon wafers’ thickness could be a viable path to further reduce production costs. In an insightful study by Liu et al. [124], the impact of silicon thickness reduction in photovoltaic systems on market expansion is analyzed. This research reveals that adopting advanced technologies with effective surface passivation can achieve comparable efficiencies between 50 μm and 160 μm Si wafers. Economically, thinner wafers are advantageous in terms of the manufacturing capital expenditure, module cost, and levelized cost of electricity, particularly for utility-scale photovoltaic systems [125,126]. Further thinning of wafers could significantly reduce costs, making the technology more economically competitive, with comparable efficiencies to conventional silicon systems. The limited adoption of thin silicon technology in photovoltaic devices, despite its potential benefits, is due to several factors. Firstly, drastic shifts in production methodologies introduce significant inertia. Secondly, there is a concern about a reduced yield in production [126]. Handling thin silicon wafers during device assembly is challenging, as their fragility leads to a significant breakage rate. This not only impacts the overall yield but also adds to the complexity and cost of the manufacturing processes in photovoltaic production. Thirdly, there is a potential loss in device efficiency, a critical factor for market competitiveness [127]. This efficiency reduction is attributed to the incomplete absorption of photons, particularly in the near-infrared region, due to the reduced thickness of the semiconductor. Silicon, with its indirect bandgap, requires a relatively long optical path to effectively absorb near-bandgap photons. This issue necessitates innovative solutions to enhance photon absorption without compromising the benefits of using thinner wafers. In this regard, two promising approaches have been developed: (i) the use of black silicon with nano-scale texturing [128] and (ii) the innovation in encapsulation materials [68]. Finally, substantial costs are associated with altering existing manufacturing and processing techniques [126,129]. These challenges create a complex landscape for the implementation of thin silicon technology, requiring not just technological solutions but also economic and strategic planning.

Organic photovoltaic materials (OPVs) exhibit potential advantages over their inorganic counterparts due to several factors such as the ease of processing, tunability of optoelectronic properties, and inherent material flexibility allowing for adaptation to different surfaces and shapes [130]. These materials have seen significant progress in efficiency, approaching a 20% threshold, which is primarily due to technological advancements like the development of new materials and alternative fabrication techniques [130]. However, achieving commercial viability for OPVs presents notable challenges, especially in maintaining efficiency and stability during large-scale production, alongside the issue of high production costs [124]. A key limiting factor for OPVs is their lower thermal and photochemical stability compared to inorganic semiconductors [124]. This reduced stability is often attributed to the presence of highly reactive double bonds and weaker intermolecular forces [85]. Despite these limitations, substantial efforts have been dedicated to the development of OPVs. This includes optimizing new material designs and investigating the photophysical processes that underlie their efficiency. Understanding these processes is crucial, as it informs strategies for enhancing their stability and performance, which are imperative for their practical application. This ongoing research and development are pivotal in overcoming the challenges faced by OPVs and unlocking their full potential in various applications, from consumer electronics to large-scale renewable energy solutions. Despite the limitations of OPVs, there is an anticipation that in the near future, billions of devices could be interconnected through the “Internet of Things” (IoT) [131]. For this interconnected network, OPVs, with their rapid response and relatively low energy production, could be ideal. They offer the potential for seamlessly integrating energy-harvesting capabilities into a myriad of devices and surfaces, facilitating a widespread, energy-efficient IoT network. The development of such systems would significantly advance the implementation of the IoT in everyday applications, revolutionizing how devices communicate and operate within this interconnected framework.

In the realm of advancing photovoltaic technologies, significant strides have been made in the commercial viability and scalability of perovskite materials. Different efforts have been primarily focused on the large-area coating of perovskites and the fabrication of high-efficiency solar modules. The latest efficiency benchmarks, as indicated in the solar cell efficiency table (version 62) [132], demonstrate the practicality of these materials in real-world applications. A notable power conversion efficiency (PCE) of 24.35% was achieved for a module with an area of 1 cm^2^, and a substantial efficiency of 22.4% was recorded for a larger module spanning 26 cm^2^ [132]. These figures underscore the potential of perovskites in scalable solar energy solutions.

The transition from laboratory-scale experiments to large-scale applications necessitates a re-evaluation of the standard practices that are used in perovskite film formation. Typically, polar aprotic solvents such as dimethyl sulfoxide or N,N-dimethylformamide (DMF), which are effective in small-scale spin-coating procedures, face challenges when applied to larger areas [132]. Their slow evaporation rates and strong interaction with components like Lewis acidic PbI_2_ in the coating solution can lead to non-uniform films, a significant barrier to scaling up production. To address these challenges, the chemistry of precursors has been adjusted, prioritizing solvents that offer a balance between the evaporation rate and interaction with perovskite constituents. Innovations in precursor engineering have led to the development of coating solutions that are suitable for large-area applications. For instance, the use of acetonitrile or 2-methoxyethanol as a solvent has shown promising results in maintaining the uniformity and quality of perovskite films on larger substrates [133]. This advancement is critical, considering that the quality of the perovskite layer directly influences the efficiency and durability of photovoltaic modules. Moreover, the introduction of cluster forms of perovskites and the strategic use of lead acetate as a kinetic controller have further enhanced the film quality and, consequently, the overall performance of perovskite-based solar cells [134]. These developments not only mark a significant step towards the commercialization of perovskite photovoltaic materials but also highlight the scalability of these technologies. The ability to produce high-quality perovskite films over large areas efficiently paves the way for their widespread adoption in the solar energy sector. This scalability is vital for meeting the growing demands for renewable energy sources and positions perovskite materials as a key player in the future of solar technology.

### 8.3. Environmental Impact

The environmental impacts of organic, silicon, and perovskite photovoltaics are diverse and significantly influence sustainable energy solutions. These materials, each at the forefront of solar technology, present unique advantages and challenges. A comprehensive understanding of their environmental impact requires examining the entire life cycle, including their manufacturing processes, energy efficiency, recycling potential, and overall sustainability.

Silicon-based photovoltaics, being the most prevalent solar technology, have undergone considerable advancements to mitigate their environmental impact, especially in manufacturing. Recent studies have focused on the energy-intensive nature of silicon photovoltaic production. For instance, Zhang et al. [135] emphasized the need to minimize environmental impacts in thin-film silicon photovoltaic production, advocating for integrated facilities and scenario functionality as key solutions. Maalouf’s research also highlighted that multicrystalline silicon demonstrates higher environmental impacts compared to thin-film technologies [136]. Jia et al. [137] further suggested that advancements in manufacturing technology and the adoption of less harmful materials can diminish the environmental effects of PV power plants, like CO_2_ emissions and land use. Important strides in sustainability are also seen in the recycling efforts of silicon-based photovoltaics. Ziemińska-Stolarska et al. [138] noted that recycling specific elements in silicon-based modules can reduce the total carbon footprint, marking significant progress in the industry’s sustainability. Liang and You [139] added that localizing silicon PV manufacturing (reshoring) contributes to decarbonization by lowering greenhouse gas emissions and energy consumption. The environmental impacts of wide-bandgap materials such as Silicon Carbide (SiC) and Gallium Nitride (GaN), as discussed by S. Glaser et al. [140], are areas that require further exploration.

In the realm of organic photovoltaic materials, recent research has underscored the development of innovative materials and processing techniques. Yu-Wei Su et al. [141] discuss advancements such as sequential deposition and layer-by-layer methods that enhance power conversion efficiency and expand potential applications, including in agriculture and greenhouses. The integration of organic photovoltaic systems into buildings, as explored by Jānis Kramens et al. [142], suggests that these systems may offer more sustainable solutions for single-family buildings, particularly in reducing particulate matter formation and global warming impacts. Recycling in the organic photovoltaic sector, emphasized by Ziemińska-Stolarska et al. [138], plays a crucial role in diminishing the environmental load, with a significant focus on effective recycling and recovery methods. In additional research conducted by Solak et al. [143], it is emphasized that organic photovoltaics represent a clean and eco-friendly technology with significant potential to reduce greenhouse gas emissions. This characteristic positions them as a viable and sustainable alternative to fossil fuel-based energy sources. Solak’s study highlights the environmental benefits of integrating organic photovoltaics in the pursuit of reducing our carbon footprint and advancing towards greener energy solutions.

Regarding the third type of materials that are studied in this review, perovskites are emerging as an efficient alternative to traditional photovoltaics. Leccisi and Fthenakis [144] conducted a comprehensive life cycle analysis comparing perovskite PV systems with crystalline–silicon and thin-film PV, finding that perovskites, especially those produced via roll-to-roll printing, have a lower environmental impact and comparable energy return on investment to single-crystalline–silicon PV within 12 years. Interfacial engineering in perovskite cells, crucial for addressing degradation from external factors, was a key focus of Kymakis’s work [107]. The environmental sustainability of perovskite PV systems, as discussed by Weyand et al. [145], hinges on achieving a five-year lifetime to qualify as a climate-friendly technology globally. In another study, Tan et al. [146] emphasized the importance of life cycle sustainability assessment in supporting the sustainable development of solar power generation, including perovskite solar cells. The recycling of perovskite solar cells, as shown by Ziemińska-Stolarska et al. [138], can reduce the environmental load significantly, highlighting the importance of sustainable end-of-life management. In line with these developments, McCalmont et al. [147] demonstrated the economic viability of recycling perovskite photovoltaics, suggesting a shift towards more sustainable practices in the industry. This research underscores the potential for perovskite photovoltaics not only in terms of efficiency and performance but also in terms of their lifecycle management, advocating for environmentally responsible and economically feasible recycling methods. Lastly, Leccisi et al. [144] also discuss the “Direct Wafer” technology in perovskite solar cells, showing notable reductions in their energy demand and global warming potential, indicative of more sustainable production methods.

The sustainability of photovoltaic technologies, including silicon, organic, and perovskite photovoltaics, has been analyzed using life cycle assessment methodologies. Urbina’s study [148,149] finds that solar electricity has lower impacts than fossil fuel electricity in various categories, emphasizing the need to consider potential risks arising from mineral scarcity in certain technologies. Another relevant aspect is the environmental impact of the materials that are used in modern solar panels, such as transparent conductive materials, encapsulation polymers, and antireflective coatings, as discussed by Dallaev et al. [150].

In conclusion, the aspects that affect the environmental impact and potential recyclability of photovoltaic materials that are used in photovoltaic systems constitute a complex and evolving field. Continuous advancements and innovations are shaping a more sustainable future for solar energy. It is crucial to understand and address the environmental implications of these technologies for their long-term viability and effectiveness in contributing to global sustainability goals. These environmental aspects must be developed in tandem with current systems and with new materials that will be developed and employed in this technology, ensuring a harmonious and sustainable advancement in the field of photovoltaics.

## 9. Innovations on the Horizon: Next-Generation Photovoltaic Materials

Silicon has long been the dominant material in photovoltaic technology due to its abundant availability and well-established manufacturing processes. As the second most common element in the Earth’s crust, silicon’s natural abundance and mature processing techniques have made it the go-to choice for solar cell production for decades. However, despite its advantages, silicon’s limitations in terms of efficiency and the complexities that are involved in its production have paved the way for exploring alternative materials. This shift is propelled by the need for more efficient energy conversion and the urge to reduce the environmental and economic costs that are linked to silicon PV technology.

The emergence of materials like perovskites, organic photovoltaics (OPVs), and quantum dots marks a transformative phase in solar technology, promising a future where solar cells are not just more efficient, but also more adaptable, lightweight, and environmentally friendly.

Perovskites: Perovskite materials have rapidly gained attention in PV research due to their impressive power conversion efficiencies, jumping from 3.8% in 2009 to over 25% recently. Their rapid advancement reflects their unique properties and the intense research interest that they have attracted. Perovskites offer low production costs and easy fabrication methods, making them economically attractive. These materials can be produced using simple techniques like solution processing, which are cheaper and less energy-intensive than traditional silicon methods. Perovskites can also be tailored at the molecular level to optimize light absorption, a crucial aspect in enhancing solar cell efficiency. When used in tandem solar cell architectures, layering them with silicon or other photovoltaic materials, they have the potential to exceed the efficiency limits of single-junction solar cells, making them a promising option for next-generation solar technologies [151,152,153,154].

Organic Photovoltaics (OPVs): OPVs signify a major shift in PV technology, being composed of carbon-based materials. These materials offer unique benefits like mechanical flexibility, leading to innovative applications such as foldable solar panels and integration into fabrics. The potential for transparent and colored solar cells opens up exciting possibilities for architectural design. While the current efficiencies of OPVs are lower than those of silicon, ongoing research is continually improving their light absorption and charge transport properties, enhancing their performance [155].

Quantum Dots: Quantum dot solar cells utilize tiny semiconductor particles whose bandgap is tunable based on their size. This property allows them to absorb different wavelengths of light more efficiently than traditional materials. Quantum dots can be engineered to create multi-junction solar cells that are capable of absorbing a wider spectrum of sunlight, potentially achieving much higher efficiencies than current single-junction cells. An intriguing application of quantum dots is in “spray-on” solar cells, which could revolutionize solar cell deployment by allowing photovoltaic materials to be applied to various surfaces, including buildings, vehicles, and clothing [151,156]

In addition to these new materials, advancements in solar technology include tandem solar cells, building-integrated photovoltaics (BIPVs), and concentrated photovoltaic systems (CPV). Tandem solar cells represent a cutting-edge approach in the field of photovoltaics, aiming to surpass the efficiency limits of single-junction solar cells [157]. By combining layers of different photovoltaic materials, each tuned to absorb a specific segment of the solar spectrum, tandem cells can convert a broader range of the solar spectrum into electrical energy. This composition often involves inorganic/organic, inorganic/perovskite, and organic/perovskite combinations, each offering unique synergistic benefits [158].

The rationale behind tandem solar cell technologies, particularly those combining inorganic/organic, inorganic/perovskite, and organic/perovskite materials, is grounded in the quest to overcome the inherent efficiency limitations of single-junction solar cells. Single-junction cells, constrained by the Shockley–Queisser limit, can only convert a fraction of the solar spectrum to electricity due to their fixed bandgap, which limits the range of photon energies that they can effectively absorb. Tandem solar cells, by contrast, are engineered to harness a broader spectrum of solar radiation by stacking multiple photovoltaic layers, each with a different bandgap, in a single device [157,158]. This configuration allows each layer to absorb different segments of the solar spectrum more efficiently than a single-junction cell could. The ultimate goal is to maximize the solar energy conversion efficiency, making solar power a more viable and competitive source of renewable energy.

Inorganic/organic tandem solar cells combine the high stability and efficiency of inorganic materials with the flexibility and tunable absorption spectra of organic compounds [158]. Inorganic materials, such as silicon or gallium arsenide, have well-established manufacturing processes and excellent electrical properties but are limited by their rigidity and high manufacturing costs. Conversely, organic photovoltaic (OPV) materials offer advantages in terms of mechanical flexibility and lower production costs, but they often suffer from lower efficiency and stability compared to their inorganic counterparts [158].

The integration of inorganic and organic layers in a tandem configuration allows for the harvesting of sunlight over a wider spectral range [159]. The inorganic layer typically absorbs the high-energy part of the spectrum, while the organic layer is optimized for lower-energy photons. This complementary absorption can significantly increase the overall efficiency of solar cells. A notable example is the tandem cell that combines a silicon bottom cell with a top cell made of organic semiconductors, aiming to overcome the Shockley–Queisser limit for single-junction cells [159].

The combination of inorganic materials with halide perovskites in tandem solar cells has emerged as a particularly promising strategy. Halide perovskites are known for their excellent optoelectronic properties, including high absorption coefficients, tunable bandgaps, and long carrier diffusion lengths [160]. When paired with traditional inorganic semiconductors, such as silicon, perovskite layers can be engineered to absorb the visible part of the solar spectrum, while the inorganic layer captures the near-infrared part [160].

This configuration not only boosts the cell’s efficiency beyond the limits of single-junction devices but also leverages the maturity and robustness of inorganic solar cell technology. For instance, a silicon–perovskite tandem cell can significantly exceed the efficiency of standalone silicon cells, with reported efficiencies exceeding 25% [161]. These tandem cells benefit from the low cost and high-efficiency potential of perovskites, coupled with the durability and established manufacturing ecosystem of silicon [161].

Tandem cells combining organic and perovskite materials bring together the best of two worlds: the mechanical flexibility and solution-processability of organic photovoltaics with the high efficiency and broad absorption spectrum of perovskite materials [162]. Organic materials can be customized to absorb specific parts of the solar spectrum that are not efficiently captured by perovskite layers, thereby complementing each other to enhance the overall device performance [162].

The future of photovoltaic materials is deeply connected to interdisciplinary research, where the fusion of material science, nanotechnology, and engineering is vital to surpass current limitations and fully realize the potential of these technologies. Exploring hybrid systems combining different types of solar cells and developing novel nanostructured materials are crucial in advancing the field. Furthermore, the sustainability of these technologies is paramount, with an emphasis on recyclability and environmentally friendly production processes to ensure the sustainable growth of solar technology.

The outlook for photovoltaic materials Is both dynamic and full of promise. As we venture into the next era of materials and technologies, the focus is firmly on boosting efficiency, curbing costs, and unveiling novel applications. This progress is poised to make solar power not only more accessible but also a seamlessly integrated part of everyday life. The fusion of cutting-edge materials with advanced technologies is at the forefront of driving the solar energy revolution, playing a crucial role in steering us towards a future that is anchored in sustainable and renewable energy. This transformative phase in photovoltaic materials is a pivotal move towards fulfilling global energy needs in a manner that is both sustainable and environmentally conscious, heralding a new chapter in the utilization of solar energy.

## 10. Conclusions

Silicon solar cells, which currently dominate the solar energy industry, are lauded for their exceptional efficiency and robust stability. These cells are the product of decades of research and development, leading to their widespread adoption in different solar applications. However, despite their technological maturity, silicon cells still grapple with economic challenges. The key lies in optimizing their production costs without compromising their performance, a task that demands innovative approaches in manufacturing techniques and material sourcing. Organic solar cells, on the other hand, present a fascinating contrast. They are celebrated for their versatility in production and the potential for reduced manufacturing costs, primarily due to their lightweight, flexible nature, and compatibility with roll-to-roll fabrication processes. This makes them particularly suitable for applications where traditional rigid panels are impractical. Nonetheless, these organic variants face significant obstacles in terms of efficiency and longevity. Their relatively lower efficiency rates, coupled with a susceptibility to degradation, underscore the need for continued research into novel organic photovoltaic materials and protective coatings that can extend their operational lifespan. Perovskite solar cells have emerged as a disruptive technology in the realm of solar energy. Characterized by their high efficiency and relatively simple fabrication process, they stand as a promising alternative to conventional photovoltaics. The remarkable progress in perovskite cell efficiency within a short period has generated considerable excitement in the research community. However, issues related to the stability of these cells, particularly under environmental stressors such as moisture and temperature fluctuations, pose significant challenges. Moreover, the environmental impact of perovskite cell materials, some of which may contain lead, raises concerns that necessitate the exploration of eco-friendly alternatives and recycling strategies.

In addressing these diverse challenges, the paper underscores the imperative for innovative research. Advancing solar energy technologies towards the pinnacles of sustainability, efficiency, and economic viability requires a multifaceted approach. It calls for concerted efforts in improving material properties, such as enhancing light absorption and charge transport mechanisms, boosting the development of novel materials, and refining existing ones. Simultaneously, it is critical to enhance manufacturing processes, making them more efficient, scalable, and environmentally benign. This includes exploring low-cost, high-throughput manufacturing techniques and developing sustainable supply chains. Furthermore, ensuring environmental safety is paramount. This involves not only minimizing the ecological footprint of solar cell production but also addressing the lifecycle impacts of these technologies, including end-of-life management and recycling. Research in this domain should aim at developing solar cells with a minimal environmental impact, from cradle to grave.

In conclusion, this review paints a comprehensive picture of the current state and future directions of solar energy development. It calls for a balanced focus on material science, engineering innovations, and environmental considerations, paving the way for a future where solar energy is not only technologically advanced but also economically feasible and environmentally sustainable. This holistic approach will undoubtedly shape the trajectory of solar energy development in the years to come, playing a crucial role in the global transition to renewable energy sources.

## Figures and Tables

**Table 1:** Comparative analysis of silicon-based, organic, halide perovskite, CIGS, CdTe, and GaAs solar cells: advantages, disadvantages, important milestones, efficiency range, applications, and environmental impact.

Solar Cell Type	Advantages	Disadvantages	Important Milestones	Efficiency Range	Applications	Environmental Impact
Silicon Solar Cells	High efficiency, durability, well-established technology	Expensive, rigid, high energy consumption	First commercial cell in 1954, efficiency over 25%	~15–25%	Residential, commercial, utility-scale solar	High carbon footprint in production
Organic Solar Cells	Flexible, low production costs, lightweight	Lower efficiency and stability, degradation under sunlight	Conductive polymers in 1977, efficiency around 10%	~3–12%	Wearable electronics, building-integrated photovoltaics (BIPVs)	Lower environmental impact, concerns about long-term waste
Halide Perovskite Solar Cells	High efficiency potential, broad absorption, tunable bandgap	Stability and toxicity concerns, scalability challenges	Introduced in 2009, efficiencies above 25%	Up to 25.5%	Potential for multi-junction solar cells, BIPVs	Toxicity, particularly from lead
CIGS Solar Cells	Good efficiency, flexible applications, less toxic	Complex manufacturing, scarcity of indium, toxicity of selenide	First report in 1975, efficiencies over 22%	~10–22%	Portable power, BIPV, space vehicles	Concerns about indium scarcity, selenide toxicity
CdTe Solar Cells	Low-cost production, good efficiency	Toxicity of cadmium, scalability and disposal issues	Developed in the 1950s, commercialized in the 1980s, efficiencies over 22%	~10–22%	Large-scale solar farms, industrial applications	Cadmium toxicity, recycling challenges
GaAs Solar Cells	Very high efficiency, excellent low-light performance, high radiation resistance	High production cost, manufacturing complexity, niche market application	Invented in 1957, efficiencies over 29%	>29%	Space applications, high-performance electronics	Costly production, arsenic toxicity concerns

## Data Availability

The data are contained in the article and are available from the corresponding authors on reasonable request.

## Abbreviations

BHJ	Bulk hetero-junction
BIPVs	Building-integrated photovoltaics
c-Si	Crystalline silicon
CPV	Concentrated photovoltaic system
CIGS	Copper indium gallium sulfur/selenide
CSPC	Carrier-selective passivating contact
Cz-Si	Czochralski-grown silicon
DST	Double-side textured
EQE	External quantum efficiency
ETL	Electron-transporting layer
FTO	Fluorine-doped tin oxide
HTL	Hole-transporting layer
IBC	Interdigitated back contact
IoT	Internet of Things
LCA	Life cycle assessment
MOF	Metal–organic framework
NBG	Narrow bandgap
OPV	Organic photovoltaic cell
OSC	Organic solar cell
PCE	Power conversion efficiency
PERC	Passivated emitter and rear cell
PID	Potential–induced degradation
PSC	Perovskite solar cell
PSM	Perovskite solar module
R2R	roll-to-roll
S2S	sheet-to-sheet
S-Q	Shockley–Queisser
SRH	Shockley–Read–Hall
SST	Single-sided textured
TSC	Tandem solar cell
TOPCon	Tunnel oxide passivated contact
WBG	Wide bandgap
